# Plasma miR-9-3p and miR-136-3p as Potential Novel Diagnostic Biomarkers for Experimental and Human Mild Traumatic Brain Injury

**DOI:** 10.3390/ijms22041563

**Published:** 2021-02-04

**Authors:** Shalini Das Gupta, Robert Ciszek, Mette Heiskanen, Niina Lapinlampi, Janne Kukkonen, Ville Leinonen, Noora Puhakka, Asla Pitkänen

**Affiliations:** 1 A.I. Virtanen Institute for Molecular Sciences, University of Eastern Finland, P.O. Box 1627, 70211 Kuopio, Finland; shalini.gupta@uef.fi (S.D.G.); robert.ciszek@uef.fi (R.C.); mette.heiskanen@uef.fi (M.H.); niina.lapinlampi@uef.fi (N.L.); noora.puhakka@uef.fi (N.P.); 2 Department of Neurosurgery, Kuopio University Hospital, 70029 Kuopio, Finland; jannekuk@student.uef.fi (J.K.); ville.leinonen@kuh.fi (V.L.); 3 Institute of Clinical Medicine, University of Eastern Finland, 70210 Kuopio, Finland

**Keywords:** biomarker, microRNA, mild TBI, miR-9-3p, miR-136-3p, plasma, severe TBI

## Abstract

Noninvasive, affordable circulating biomarkers for difficult-to-diagnose mild traumatic brain injury (mTBI) are an unmet medical need. Although blood microRNA (miRNA) levels are reportedly altered after traumatic brain injury (TBI), their diagnostic potential for mTBI remains inconclusive. We hypothesized that acutely altered plasma miRNAs could serve as diagnostic biomarkers both in the lateral fluid percussion injury (FPI) model and clinical mTBI. We performed plasma small RNA-sequencing from adult male Sprague–Dawley rats (*n* = 31) at 2 days post-TBI, followed by polymerase chain reaction (PCR)-based validation of selected candidates. miR-9a-3p, miR-136-3p, and miR-434-3p were identified as the most promising candidates at 2 days after lateral FPI. Digital droplet PCR (ddPCR) revealed 4.2-, 2.8-, and 4.6-fold elevations in miR-9a-3p, miR-136-3p, and miR-434-3p levels (*p* < 0.01 for all), respectively, distinguishing rats with mTBI from naïve rats with 100% sensitivity and specificity. DdPCR further identified a subpopulation of mTBI patients with plasma miR-9-3p (*n* = 7/15) and miR-136-3p (*n* = 5/15) levels higher than one standard deviation above the control mean at <2 days postinjury. In sTBI patients, plasma miR-9-3p levels were 6.5- and 9.2-fold in comparison to the mTBI and control groups, respectively. Thus, plasma miR-9-3p and miR-136-3p were identified as promising biomarker candidates for mTBI requiring further evaluation in a larger patient population.

## 1. Introduction

Traumatic brain injury (TBI) is defined as “an alteration in brain function, or other evidence of brain pathology, caused by an external force” [1]. Each year, approximately 69 million people worldwide suffer TBI, with mild TBI (mTBI) accounting for approximately 80% to 90% of those cases [2]. mTBI presents a particular diagnostic challenge because a large majority of patients do not exhibit visible structural damage to the brain detectable by the current standard imaging methods that are widely available in trauma centers (e.g., computed tomography) [3]. This necessitates the use of functional and metabolic imaging modalities to diagnose molecular-level pathobiological changes ongoing in the brain post-mTBI [4]. Even in the absence of a visible structural damage to the brain, patients with mTBI may still suffer from long-term neurologic and psychiatric impairments, compromising their ability to work and diminishing their overall quality-of-life [5]. Thus, there is a major unmet medical need to identify accurate, affordable, and widely accessible diagnostic biomarkers to guide symptomatic and rehabilitative treatments of mTBI patients.

Blood-based biomarkers are minimally invasive, cost-effective, and hold promise for characterizing TBI severity and providing molecular-level information about the ongoing pathologic changes in different neuronal cell types [6]. Diagnosis of mTBI using blood biomarkers can further facilitate follow-up care as well as prognosis of chronic recovery or adverse postinjury outcomes [7,8,9,10]. To date, only serum glial fibrillary acidic protein (GFAP) and ubiquitin *C*-terminal hydrolase L-1 (UCHL1) tests have been approved as biomarkers for mTBI by the United States Food and Drug Administration [11]. Although the sensitivity and negative predictive values of these biomarkers are approximately 98% to 100%, respectively, the maximum specificity and positive predictive values are approximately only 37% and 10%, respectively. Other plasma/serum proteins like S100B have also been used as research tools in conjunction with other measures for diagnosing mTBI but circulating S100B levels are also altered as a result of peripheral injury, thereby decreasing the sensitivity of S100B for mTBI diagnosis [12,13,14,15]. Thus, the identification of circulating brain-derived biomarkers that are both sensitive and specific for mTBI is needed.

MicroRNAs (miRNAs) are endogenous small noncoding RNAs (~22 nucleotides long) that regulate the expression of protein-coding genes through translational repression [16,17]. The levels of circulating miRNAs have been investigated in human TBI patients, but very few are reported to be consistently altered by TBI [18,19,20,21,22,23,24,25,26]. Animal models of TBI are crucial for biomarker identification as they provide homogenous and reproducible methods for blood biomarker discovery, avoiding several confounding factors that contribute to the heterogeneity of the TBI patient population [6,27,28]. Thus far, only three studies have investigated the levels of circulating miRNAs as biomarkers of TBI in animal models [29,30,31], and only one group has investigated the dependency of serum and brain miRNA levels on impact severity [31,32]. In those studies, however, the sensitivity and specificity of the candidate miRNAs were not assessed.

Thus, the objective of the present study was to identify sensitive and specific diagnostic plasma miRNA biomarkers for mTBI in a clinically relevant animal model of TBI and to analyze the translational potential of these candidate biomarkers in a TBI patient population. Further, our objective was to identify if the miRNA biomarkers would exhibit an impact-severity-dependent increase in plasma levels, both in the experimental lateral FPI model and in human patients with mild (mTBI) and severe TBI (sTBI).

## 2. Materials and Methods

### 2.1. Animals

Adult male Sprague–Dawley rats (*n* = 33, body weight 354–392 g at the time of injury, Envigo B.V., Horst, Netherlands) were used. The rats were housed in a controlled environment (temperature 22 ± 1 °C; humidity 50–60%; lights on from 07:00 to 19:00 h). Water and pellet food were provided ad libitum.

### 2.2. Induction of TBI with Lateral Fluid-Percussion

The rats were subjected to lateral FPI-induced TBI (mTBI: 10 rats, sTBI: 10 rats) as described previously [33,34,35]. Briefly, the animals were anesthetized by inhalation of 5% isoflurane (room air as carrier gas) and placed in a Kopf stereotactic frame (David Kopf Instruments, Tujunga, CA, USA). During the surgery, anesthesia was maintained with 2% isoflurane through a nose cone (room air as carrier gas). The rats were kept on a heating pad during the surgery and body temperature was recorded using a rectal probe. Lidocaine (200 µL, 5 mg/mL, Orion Pharma, Finland) was injected subcutaneously (s.c.) over the planned incision area. A midline scalp incision was then made, and the underlying periosteum was removed. A circular craniectomy (diameter 5 mm) was performed with a trephine over the left hemisphere midway between lambda and bregma, with the lateral edge of the craniectomy adjacent to the lateral ridge (craniotomy center ML 2.5 mm and AP −4.5 mm from bregma). A modified Luer–lock cap was placed into the craniectomy and its edges were sealed with glue. Then, the cap was cemented onto the skull (Selectaplus CN, Dentsply DeTRey GmbH, Dreieich, Germany) and filled with saline. After completion of the surgery, the rats were removed from the stereotax and the toe-pinch reflex was monitored. At the first response to toe-pinch, they were connected to the fluid-percussion device equipped with a straight tip (AmScien Instruments, Richmond, VA, USA) through the male Luer–lock fitting, and the brain injury was induced (mTBI: 1.5 ± 0.1 atm, sTBI: 2.9 ± 0.1 atm). Impact severity was assessed based on acute postimpact mortality (<72 h post-TBI), duration of postimpact apnea, time to regain righting reflex and Nissl staining of the brain tissue. Sham-operated control rats (*n* = 8) received anesthesia and underwent all surgical procedures without lateral FPI. A group of 5 naïve rats was also included. The naïve rats were anesthetized with 5% isoflurane for 5 min on the day of TBI induction and returned to their cages without undergoing any surgical procedures.

Postimpact monitoring of animal well-being was performed as described previously [36]. Immediately postimpact or postsurgery, the rats (TBI and sham-operated) were removed from the device and placed on a heating pad. Time in apnea and the time to righting were monitored. After the animals righted, they were administered with 10 mL of 0.9% saline (s.c.). They also received 0.05 mg/kg (s.c.) buprenorphine (Orion Pharma, Finland) for postoperative analgesia. To minimize suffering and distress, overall well-being of the rat and its motor activity, eating and drinking were observed daily until sacrifice. The rats with sTBI received soft powdered pellet food and water from a serving dish placed on the floor of the cage for two days postinjury. All TBI-induced rats received 10 mL of 0.09% saline subcutaneously (1–2 times/day) at 1 d postinjury. All animals were weighed at baseline (7 d or 14 d prior to injury), on the day of surgery/TBI, and then daily for the following two days.

Based on previous experience with the model, we expected <10% mortality due to anesthesia or mild TBI impact. A 20–30% acute postimpact mortality (within 72 h) was expected for the severe TBI impact group.

### 2.3. Sampling of Plasma and Brain Tissue

Tail-vein plasma was sampled from the rats (*n* = 31) at 2 days post-TBI, according to the principles of 3Rs (www.nc3rs.org.uk/rat-tail-vein-non-surgical; (accessed on 15 September 2019)) and our previously optimized protocols [37,38]. Briefly, whole blood was drawn from the lateral tail-vein under isoflurane anesthesia (induction: 5%, maintenance: 2%), into Microtainer K_2_ EDTA-tubes (di-potassium ethylenediaminetetraacetic acid, Microtainer, BD Biosciences, Franklin Lakes, NJ, USA), using a 25G butterfly needle (Surflo Winged infusion set, Terumo Europe N.V., Leuven, Belgium). At 2 days post-TBI, 2 mL of whole blood was collected into four EDTA tubes (EDTA A-D, 500 μL/tube). The whole blood samples were thoroughly mixed with EDTA and placed on ice. Centrifugation was performed immediately at 1300× *g* (Centrifuge 5417R, Eppendorf Biotools, CA, USA) for 10 min (+4 °C) to obtain plasma. Hemolysis coefficient of the plasma was measured from each EDTA tube at 414 nm (UV-Vis module) using a NanoDrop-1000 spectrophotometer available in our laboratory, prior to aliquoting in 50 µL aliquots (Protein LoBind tubes, Eppendorf LoBind, Eppendorf AG, Hamburg, Germany). The aliquots were frozen in dry ice and stored at −70 °C until processed.

Immediately after tail-vein blood sampling at 2 days post-TBI, the rats were perfusion-fixed and their brain tissue was processed for histology to assess the lesion location and injury severity as described previously [30]. For this, the rats were deeply anesthetized with 5% isoflurane and decapitated. The brains were quickly removed and immersion-fixed in 10% buffered formalin for 2–3 days. This was followed by cryoprotection in 20% glycerol in 0.02 M potassium phosphate buffered saline (pH 7.4). The brains were then stored at −70 °C until processed. For Nissl staining, the brains were sectioned in 1-in-5 series of coronal sections (30 µm) with a sliding microtome (Leica SM 2000, Leica Microsystems Nussloch GmbH, Nussloch, Germany). The first series was stored in 10% buffered formalin at room temperature and used for Nissl staining. The remaining sections were stored in tissue-collecting solution (30% ethylene glycol, 25% glycerol in 0.05 M sodium phosphate buffer, pH 7.4) at −20 °C until further processing.

### 2.4. Small RNA-Seq from Plasma

#### 2.4.1. Library Preparation and Sequencing

RNA extraction from the plasma and small RNA sequencing were performed by the Qiagen Genomics Services (Hilden, Germany; *n* = 20: 5 naïve, 5 sham-operated controls, 5 mTBI and 5 sTBI). For each animal, 5 frozen 50-µL plasma aliquots from the EDTA-A and EDTA-B tubes were shipped to the Qiagen center (Hilden, Germany), where the aliquots were thawed and pooled to obtain 250 µL plasma for each case. The plasma hemolysis coefficient was measured from the pooled plasma at 414 nm with NanoDrop. RNA was then isolated from 200 µL plasma using the miRNeasy serum/plasma kit and eluted to 14 µL. The extracted RNA was subjected to qPCR-based quality control. Small RNA library preparation was then performed with the QIASeq miRNA library kit for Illumina NGS systems (performed at the Qiagen center, Hilden, Germany). Single-end sequencing of 75-bp reads was performed at a depth of 12M, with one sample/lane in the Illumina NextSeq 550.

#### 2.4.2. Quantification of miRNAs and Differential Expression Analysis

The raw fastq files obtained from small RNA-Seq were first manually inspected with FastQC (v. 0.11.3) to check the overall quality of the sequencing data. The fastq files were then uploaded to the Qiagen Geneglobe Data Analysis Center (DAC), a freely available web resource to analyze data from Qiagen’s QIASeq NGS library kits (https://geneglobe.qiagen.com/in/analyze/; (accessed on 15 September 2019)). Primary quantification of read counts was performed in the DAC, which involved the following steps: (i) trimming of the 3′-adapter and low-quality bases using cutadapt; (ii) identifying the insert sequences and UMIs (reads with <16 bp insert sequences or <10 bp UMI sequences were discarded); (iii) alignment of the processed reads to the rat reference genome RGSC Rnor_6.0 with a sequential alignment strategy using bowtie (perfect match to miRBase mature, miRBase hairpin, noncoding RNA, mRNA and other RNA, and ultimately a second mapping to miRBase mature, where up to 2 mismatches were tolerated). Annotation of the miRNAs was performed with miRBase v. 21. Following primary quantification, differential expression analysis for miRNAs was performed with DESeq2 (v. 1.22.2) [39] in the R environment (v. 3.5.3). Data were visualized with R (v. 3.5.3).

#### 2.4.3. Identification of Expression Pattern Differences with Machine Learning

We also applied logistic regression with feature selection, utilizing nested leave-one-out CV to identify miRNAs (“features”) that contributed the most to the group differences (differences between naïve, sham, mTBI, sTBI, TBI (sTBI + mTBI), and uninjured (naïve + sham)). The logistic regression model was optimized on raw counts from miRNAs with a count ≥ 1 in at least 80% of samples to maximize separation between groups in terms of the area under the curve (AUC) of the receiver operating characteristic (ROC) curve. The miRNA counts were standardized to 0 mean and unit variance. In the inner CV loop, miRNAs with 0 variance were filtered and feature selection was performed using recursive feature elimination and filtering by F-score. Model hyperparameters and feature selection configurations were optimized with a grid search over combinations of regularization factor levels, feature selection methods, number of selected features, and choices between L1 (LASSO) and L2 (Ridge) regularization. Permutation tests were performed to assess the statistical significance of model CV AUC scores. Feature importance was calculated by averaging the absolute values of logistic regression model covariates over the outer fold of nested CV. The averaged values were normalized to sum to one and ordered in descending order to identify miRNAs that contributed the most to group separability. The analyses were performed using Python (3.7.0) and sklearn package (20.2) on Centos 7.

### 2.5. Technical Validation of Small RNA-Seq Data

#### 2.5.1. Selection of an Endogenous miRNA for Normalization of RT-qPCR

The NormFinder add-in (https://moma.dk/normfinder-software; (accessed on 15 September 2019)) was run in Microsoft Excel on the miRNA raw read count table obtained from primary quantification at Geneglobe to identify the most stable miRNAs. According to the results, miR-28-3p (stability value 732.79) was identified as the most stable endogenous miRNA in the dataset.

#### 2.5.2. RT-qPCR of Selected miRNA Candidates

Reverse transcription was performed from the same RNA samples that were used for small RNA-Seq (*n* = 19: 4 naïve, 5 sham-operated controls, 5 mTBI, and 5 sTBI). Total RNA was transcribed to cDNA with the miRCURY LNA RT Kit (Catalog No. 339340, Qiagen, Hilden, Germany) according to the manufacturer’s instructions. The 5 miRNA candidates selected for technical validation were rno-miR-9a-3p, rno-miR-153-3p, rno-miR-15a-3p, rno-miR-136-3p, and rno-miR-434-3p (see Results). According to the protocol, the cDNA samples were first diluted 30-fold with nuclease-free water. The 10 µL PCR reaction was then prepared with 5 µL 2× miRCURY SYBR Green PCR master mix, 1 µL each of the miRCURY PCR primer assays (Catalog No. YP00204620 (rno-miR-9a-3p), YP00204338 (rno-miR-153-3p), YP00205503 (rno-miR-136-3p), YP00205190 (rno-miR-434-3p), YP00204119 (rno-miR-28-3p), YCP0051329 (rno-miR-15a-3p), Qiagen) and RNase-free water, and 3 µL diluted RT product. RT-qPCR was run using a Roche LightCycler 96 Real-Time PCR System with a standard program: 95 °C for 2 min and 40 cycles of 95 °C for 10 s and 56 °C for 1 min. Data were normalized to miR-28-3p using the formula 2^−ΔCt^.

### 2.6. Analysis of Validated miRNAs in the Whole Cohort

#### 2.6.1. Plasma Hemolysis Measurement

Following technical validation from the same RNA samples that were used for small RNA-Seq, the validated miRNA candidates (rno-miR-9a-3p, rno-miR-136-3p, and rno-miR-434-3p; see Results) were analyzed from the plasma of all animals of the cohort at 2 days post-TBI (*n* = 31: 5 naïve, 8 sham-operated controls, 10 mTBI, and 8 sTBI). For this, a separate 50-µL plasma aliquot from each animal was used. Prior to RNA extraction, hemolysis of the individual aliquots was measured with a NanoDrop-1000 spectrophotometer at 414 nm.

#### 2.6.2. RT-qPCR of Validated miRNA Candidates

Total RNA was isolated using the miRNeasy serum/plasma protocol (Qiagen, Hilden, Germany, https://www.qiagen.com/fi/resources/resourcedetail?id=1076a54d-7967-4bc0-a34e-e3f574641d92&lang=en; (accessed on 15 September 2019)), with slight modifications: the addition of any spike-in control was omitted, and final elution of RNA was performed in 30 μL, rather than 14 µL, nuclease-free water. Reverse transcription was performed using the miRCURY LNA RT Kit as described above. RT-qPCR based validation was performed for the technically validated candidates (rno-miR-9a-3p, rno-miR-136-3p, and rno-miR-434-3p; see Results). Because the RNA used in this step was extracted from 50 µL plasma instead of 200 µL, and eluted to 30 µL instead of 14 µL, we used a 10-fold dilution of the cDNA instead of the 30-fold dilution used in the technical validation step, to obtain detectable levels of the miRNAs. Preparation of the RT-qPCR reaction mixture and thermal cycling conditions were the same as above. For the whole cohort, miR-28-3p was used as the endogenous control and the RT-qPCR data was normalized to it using the formula 2^−ΔCt^.

#### 2.6.3. ddPCR of Validated miRNA Candidates

The digital droplet PCR (ddPCR) method performs absolute nucleic acid quantification with greater precision and day-to-day reproducibility in comparison to real time PCR [40]. Thus, to obtain absolute copy numbers of the validated miRNA biomarker candidates, we performed ddPCR analysis from the whole animal cohort. The 20 μL ddPCR reaction mixtures were prepared with 10 μL Bio-Rad 2× ddPCR EvaGreen Supermix (#186-4034, Bio-Rad), 1 μL miRNA PCR primers, 1 μL nuclease-free water, and 8 μL diluted cDNA template (10-fold dilution for miR-9a-3p and miR-434-3p, 5-fold dilution for miR-136-3p). Droplets were generated with 70 μL of droplet generation oil for EvaGreen (#186-4005, Bio-Rad) as described previously [30]. The thermal cycling conditions for ddPCR were as follows: 95 °C for 5 min, 40 cycles of 95 °C for 30 s, and 56 °C for 1 min, 4 °C for 5 min, 90 °C for 5 min, and then maintained at 4 °C. To obtain a clear separation between the clusters of positive and negative droplets, the fluorescence amplitude threshold was manually adjusted to 10,000 for rno-miR-9a-3p and rno-miR-434-3p, and 6000 for rno-miR-136-3p for all samples. For each sample, the reaction was performed in duplicate, and the mean miRNA copy number/20-μL PCR reaction well from the 2 replicates was calculated and used for data representation in the figures.

### 2.7. Analysis of the Validated miRNAs in Plasma from Human TBI Patients

The patient characteristics are summarized in Table 1. Human mTBI plasma samples [(*n* = 15; Glasgow Coma Score (GCS) 15) were obtained from the Kuopio University Hospital. Plasma from 2 sTBI cases (GCS 3–4) was also obtained from the same hospital. The mTBI plasma samples were collected within 48 h after injury (mean 11.0 h postinjury, range 1.5–48 h). Plasma samples from age-matched controls were obtained for 12 of 15 mTBI patients and the 2 sTBI patients.

Plasma was prepared using the following protocol: 10 mL whole blood collected in 1 EDTA tube (10 mL/tube) and centrifuged immediately at 2200× *g* for 10 min at room temperature. The plasma was then aliquoted and frozen at −70 °C. Total RNA was isolated from 200 µL plasma using the miRNeasy serum/plasma protocol. The same modifications were applied as for the animal cohort. Reverse transcription was performed from all samples using the miRCURY LNA RT Kit as described for the animal cohort.

#### 2.7.1. ddPCR of Validated miRNA Candidates

To obtain absolute copy numbers for hsa-miR-9-3p and hsa-miR-136-3p, ddPCR analysis was performed. The ddPCR reaction protocol and thermal cycling conditions were the same as described previously for the animal cohort (10-fold cDNA dilution used for both miR-9-3p and miR-136-3p).

#### 2.7.2. ddPCR from Small RNA Concentration-Normalized Samples

To avoid possible bias related to variable total RNA concentrations in different samples as a result of the biologic variability between patients and technical variability introduced during the RNA extraction, we also analyzed miR-9-3p and miR-136-3p levels from the human plasma samples with the small RNA concentration-normalization approach, as described previously [41]. A reverse transcription reaction was then performed from the adjusted RNA samples using the miRCURY LNA RT kit as described previously, followed by ddPCR analysis.

### 2.8. Statistical Analysis

Differential miRNA expression analysis was performed with DESeq2. Differential expression was considered at a level of a false detection rate < 0.05. Other statistical analyses were performed using IBM SPSS Statistics 25.0 (IBM Corp., Armonk, NY, USA). Graphs were prepared with R (v3.5.3) and GraphPad Prism (v. 8.0.1, GraphPad software, San Diego, CA, USA). Comparisons of 3 or more groups were performed using the nonparametric independent samples Kruskal-Wallis test, followed by Mann–Whitney *U* test. Correlations were analyzed based on Spearman’s rho (ρ). ROC analysis was performed for each validated miRNA to investigate its sensitivity and specificity in distinguishing group differences. Statistical significance of the AUC was assessed with the Mann–Whitney *U* test. The optimal cut-off of plasma miRNA copy number separating the study groups was evaluated with the cutpointr package (v. 1.0.1) in R [42], maximizing for the sum of sensitivity and specificity, as described previously [43]. A *p*-value less than 0.05 was considered statistically significant. Data are represented as mean ± SD.

## 3. Results

### 3.1. Impact Severity, Mortality, Duration of Postimpact Apnea, and Time to Righting

Impact severity in the severe TBI (sTBI) group was 1.9-fold higher than that in the mTBI group (2.9 ± 0.1 atm vs. 1.5 ± 0.1 atm, *p* < 0.001, Figure S1A). Further, in the sTBI group, acute post-TBI mortality (<48 h) was 20% (2/10). No mortality was observed in the naïve, sham-operated control or mTBI groups. Naïve and sham-operated controls exhibited no apnea. In the mTBI group, the duration of acute postimpact apnea was 0.8 ± 1.8 s. In the sTBI group, the duration of acute postimpact apnea was 32.5 ± 17.7 s (*p* < 0.001 compared with mTBI, Figure S1B). Time to righting was 4.4 ± 2.2 min after sham surgery, 7.2 ± 1.6 min after mTBI (*p* < 0.05 compared to sham-operated controls), and 10.6 ± 5.4 min after sTBI (*p* < 0.01 compared with sham-operated controls). The righting times did not differ significantly between the mTBI and sTBI groups (*p* > 0.05) (Figure S1C).

For both the mTBI and sTBI groups, Nissl-stained brain sections showed neuronal cell loss at the lesion core in the cortex. The extent of the cell loss and hemorrhage in the perilesional cortex and underlying white matter were more prominent in the sTBI group than in the mTBI group (Supplementary Figure S2).

### 3.2. Quality Control Analysis from Tail-Vein Plasma and Extracted RNA Prior to Small RNA-Seq

#### 3.2.1. Hemolysis Measurement with NanoDrop

Plasma samples were considered hemolyzed if the hemolysis coefficient was >0.25 at 414 nm in the NanoDrop spectrophotometer [37,44]. Among the EDTA-A tubes, only 3% (1/31) were hemolyzed. None of the EDTA-B tubes contained a hemolyzed sample. Next, hemolysis in 250 µL of pooled plasma (pooled from five 50 µL aliquots of the EDTA-A and EDTA-B tubes) was measured. In the pooled plasma, 35% (7/20) of the samples were hemolyzed, 2 of which had hemolysis coefficients marginally above the threshold of 0.25. Hemolysis coefficients of the pooled plasma positively correlated with that of the individual EDTA-A (ρ = 0.652, *p* < 0.01) and EDTA-B tubes (ρ = 0.658, *p* < 0.01).

#### 3.2.2. Hemolysis Measurement with the ΔCq (miR-23a–miR-451) Method

This method detects hemolysis after RNA extraction. A ΔCq (miR-23a–miR-451) value > 5 indicates hemolysis. Using this method, none of the pooled plasma samples were hemolyzed (Figure S3A).

#### 3.2.3. Amplification of Endogenous miRNAs and Spike-In Controls

The expression levels of a panel of endogenous miRNAs with relatively abundant expression in serum and plasma (hsa-miR-103a-3p, hsa-miR-191-5p, hsa-miR-451a, hsa-miR-23a-3p, and hsa-miR-30c-5p) was analyzed. All samples had Cq values within the expected range for these miRNAs (Figure S3B). The expression levels of the spike-in controls, UniSp6, UniSp-100, and UniSp-101, were also within the expected range (Figure S3C).

### 3.3. Primary miRNA Quantification

**Figure 1:**
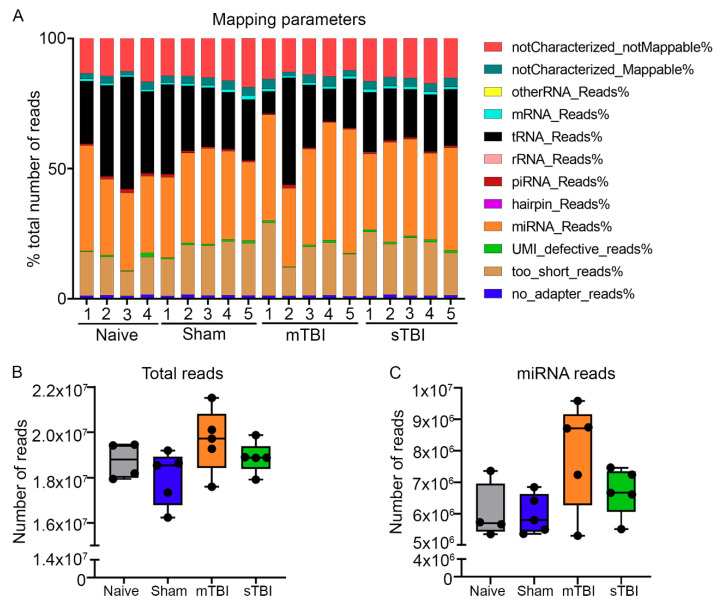
Primary quantification revealed no difference between the total sequenced reads and the sequenced reads mapping to miRNAs between experimental groups. (**A**) miRNA and transfer RNA reads comprised of 50–70% of the total sequenced reads (*y*-axis) across all samples in the experimental groups (*x*-axis; naïve *n* = 4, sham *n* = 5, mTBI *n* = 5, sTBI *n* = 5). (**B**) The total number of detected reads (*y*-axis) did not differ among the four groups (*x*-axis). (**C**) Similarly, total number of reads mapping to miRNAs (*y*-axis) also did not differ among the four groups (*x*-axis). Abbreviations: mRNA, messenger RNA; miRNA, microRNA; mTBI, mild traumatic brain injury; piRNA, Piwi-interacting RNA; rRNA, ribosomal RNA; sham, sham-operated experimental controls; sTBI, severe traumatic brain injury, tRNA, transfer RNA; UMI, unique molecular index.

**Figure 2:**
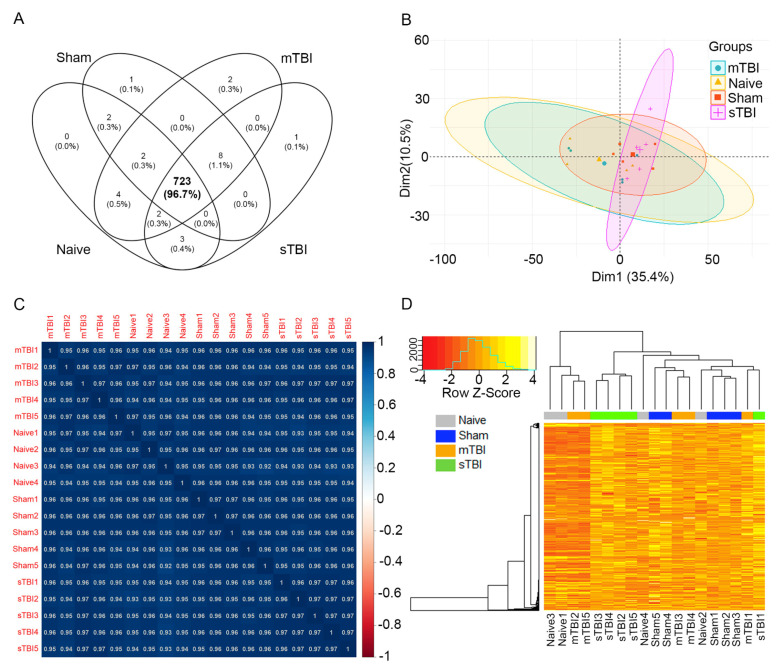
The overall miRNA expression profile in rat plasma was similar between the TBI and control groups. (**A**) Venn-diagram of micro RNAs expressed in each group (naïve, sham, mTBI, sTBI). Among the 748 miRNAs detected across all samples in miR-sequencing, 723 (97%) were commonly expressed in all 4 groups. (**B**) Principal component analysis revealed overall similarity in the miRNA expression profile in the four groups, as the first two components (Dim1 + Dim2) explained only 46% of the variance in the dataset. (**C**) High positive Spearman correlation coefficients were observed across all samples. (**D**) Heatmap based on Spearman rank correlation as the distance matrix also indicated similar miRNA expression profile across groups (no clustering of cases). Top shows the clustering of samples, left the clustering of miRNAs, and bottom the individual animals. Abbreviations: Dim1, dimension 1; Dim 2, dimension 2; mTBI, mild traumatic brain injury; sham, sham-operated experimental controls; sTBI, severe traumatic brain injury; Z-Score, measure of distance in standard deviations from the mean.

One sample from a naïve rat failed at the library preparation step. Therefore, small RNA-Seq was performed on 19 cases: 4 naïve, 5 sham-operated controls, 5 mTBI, and 5 sTBI. Primary quantification with the Geneglobe portal identified that the total sequenced reads per sample comprised the following: (1) reads without adapters; (2) too short reads (reads with <16-bp insert sequences); (3) reads with defective unique molecular index (UMIs; reads with <10 bp UMI sequences); (4) miRNA reads; (5) hairpin reads; (6) Piwi-interacting RNA reads; (7) ribosomal RNA reads; (8) transfer RNA reads; (9) mRNA reads; (10) other RNA reads; (11) mapped reads that could not be characterized as a particular type (notCharacterized_Mappable); and (12) reads that could not be mapped (notCharacterized_notMappable) (Figure 1A). A mean of 20% of reads were discarded prior to mapping in the primary quantification pipeline (reads without adapters, too short reads, and reads with defective UMIs: 11–30% of total sequenced reads). The mapping percentage across all samples was 65% (reads successfully mapped to miRNA, hairpin, Piwi-interacting RNA, ribosomal RNA, transfer RNA, mRNA, other RNA, and notCharacterized_Mappable: 54–77% of total sequenced reads). The mapping percentage specifically to miRNAs across all samples was 35% (29–48% of total sequenced reads). Across all samples, 15% of reads failed to map (notCharacterized_notMappable: 12–19% of total sequenced reads). The total number of sequenced reads did not differ between the naïve, sham-operated controls, mTBI, and sTBI groups (*p* > 0.05, Figure 1B). The number of reads mapping to miRNAs also did not differ among the 4 groups (*p* > 0.05, Figure 1C). A total of 748 miRNAs were detected across all samples, of which 723 (97%) were expressed in all 4 groups (Figure 2A).

Normalization to counts per million was performed on the raw read count data using the following Equation (1):

(1) $$\frac{read\;count\;of\;a\;given\;miRNA\;in\;a\;sample}{total\;number\;of\;reads\;mapped\;to\;miRNAs\;in\;that\;sample}\times 1,000,000$$

**Figure 3:**
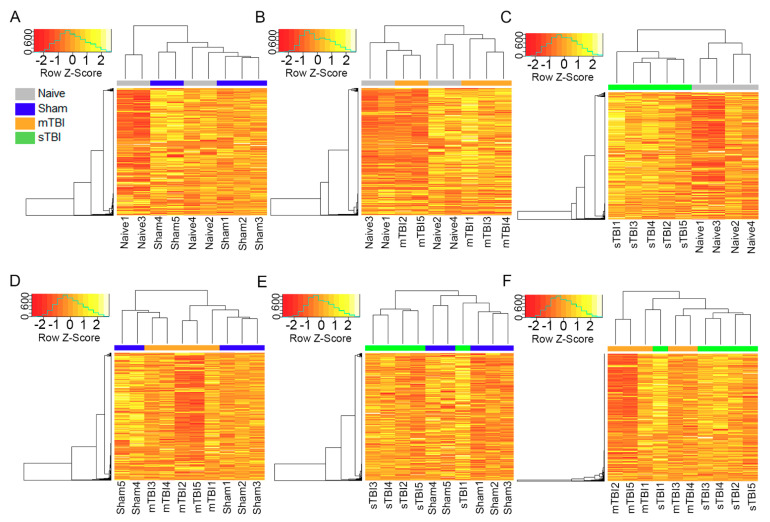
Pairwise comparison of overall miRNA expression profile in rat plasma revealed separate clustering only between the naïve and sTBI groups. When heatmaps (**A**–**F**) were plotted based on the Spearman rank correlation as the distance matrix for pairwise group comparisons, the overall miRNA expression profiles separated (**C**) the naïve and sTBI groups. No separation of clusters was found between (**A**) naïve and sham groups, (**B**) naïve and mTBI groups, (**D**) sham and mTBI groups, (**E**) sham and sTBI groups, and (**F**) mTBI and sTBI groups. In each panel, top shows the clustering of samples, left the clustering of miRNAs, and bottom the individual animals. Abbreviations: mTBI, mild traumatic brain injury; sham, sham-operated experimental controls; sTBI, severe traumatic brain injury; Z-Score, measure of distance in standard deviations from the mean.

The normalized data were visualized using principal component analysis, Spearman correlation matrices, and heatmaps with Spearman correlation as the distance measurement and complete linkage as the clustering method. The principal component analysis revealed that the first and second principal components explained 46% of the variance in the data (Dimension1 35% and Dimension2 11%, Figure 2B). High-positive Spearman correlation coefficients were obtained across all samples (Figure 2C). The heatmap of all the samples did not reflect a clear separation among the 4 groups (Figure 2D). This indicated that the overall miRNA expression profiles were very similar among the 4 groups. When pairwise comparisons of the overall miRNA expression profile were analyzed with a heatmap, a clear separation was observed between the naïve and sTBI groups (Figure 3C).

### 3.4. Differential Expression Analysis

**Figure 4:**
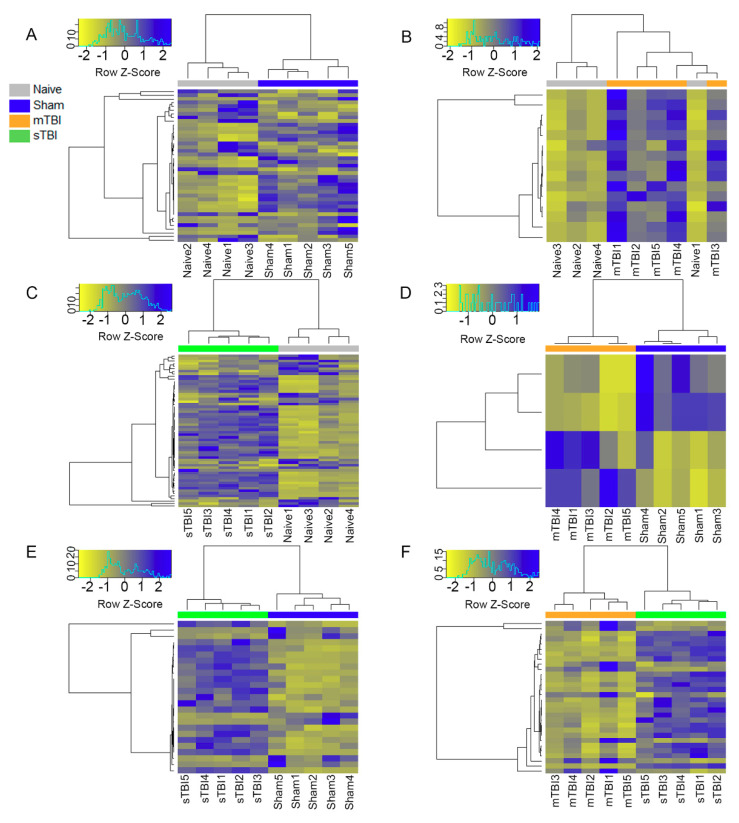
Heatmaps for differentially expressed miRNAs in rat plasma revealed separation of different groups. (**A**–**C**) In comparison to the naïve group, the sham-operated controls had 41 differentially expressed miRNAs (18 downregulated and 23 upregulated), the mTBI group 15 differentially expressed miRNAs (all upregulated), and the sTBI group 60 differentially expressed miRNAs (21 downregulated and 39 upregulated). (**D**,**E**) Compared with the sham-operated experimental controls, the mTBI group had four differentially expressed miRNAs (two downregulated and two upregulated) and the sTBI group had 25 differentially expressed miRNAs (6 downregulated and 19 upregulated). (**F**) The sTBI group had 30 differentially expressed miRNAs in comparison to the mTBI group (8 downregulated and 22 upregulated). In each panel, top shows the clustering of samples, left the clustering of miRNAs, and bottom the individual animals. Abbreviations: mTBI, mild traumatic brain injury; sham, sham-operated experimental controls; sTBI, severe traumatic brain injury; Z-Score, measure of distance in standard deviations from the mean.

Differential expression (DE) analysis was performed for each pairwise group combination. Compared with naïve rats, sham-operated controls had 41 differentially expressed miRNAs (18 downregulated and 23 upregulated, Figure 4A), the mTBI group had 15 differentially expressed miRNAs (all upregulated, Figure 4B), and the sTBI group had 60 differentially expressed miRNAs (21 downregulated and 39 upregulated, Figure 4C). Compared with sham-operated controls, the mTBI group had 4 differentially expressed miRNAs (2 downregulated and 2 upregulated, Figure 4D) and the sTBI group had 25 differentially expressed miRNAs (6 downregulated and 19 upregulated, Figure 4E). The sTBI group also had 30 differentially expressed miRNAs in comparison to the mTBI group (8 downregulated and 22 upregulated, Figure 4F).

### 3.5. Expression Pattern Differences from Machine Learning

**Figure 5:**
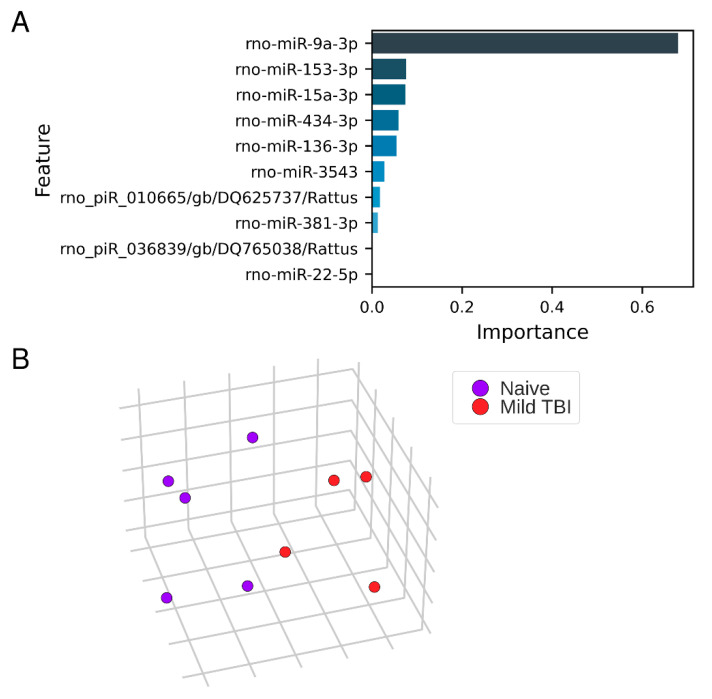
Machine learning revealed miRNAs differentiating mTBI rats from naïve animals. Feature importance (**A**) from logistic regression models optimized for naïve vs. mTBI classification. The feature importance denotes the absolute values of model coefficients averaged over CV folds and normalized to sum to 1. The majority of the coefficient mass centered on rno-miR-9a-3p, tapering to 0 after 8 top coefficient. The top five miRNA candidates with the highest feature importance distinguishing the mTBI group from the naïve rats were selected for technical validation of the small RNA-Seq data: rno-miR-9a-3p, rno-miR-153-3p, rno-miR-15a-3p, rno-miR-136-3p, and rno-miR-434-3p. (**B**) t-SNE reduction of raw counts from miRNAs with count ≥ 1 in at least 80% samples. The visible separability of the two groups explains the linear model’s ability to discern between naïve and mTBI groups.

Logistic regression analysis differentiated between naïve and sham groups with cross-validated area under the curve (CV AUC) 0.95 (*p* = 0.026), and between mTBI and naïve groups with CV AUC 1.0 (*p* = 0.059). The top 5 miRNA candidates that distinguished the mTBI group from the naive group based on the feature importance from logistic regression analysis were selected for technical validation of the small RNA-Seq data: rno-miR-9a-3p, rno-miR-153-3p, rno-miR-15a-3p, rno-miR-136-3p, and rno-miR-434-3p (Figure 5). Among these, rno-miR-9a-3p, rno-miR-136-3p, and rno-miR-434-3p were also identified by the DE analysis.

### 3.6. Technical Validation of Regulated rno-miR-9a-3p, rno-miR-153-3p, rno-miR-15a-3p, rno-miR-136-3p, and rno-miR-434-3p Levels in Samples Used for miR-Seq

Spearman analysis revealed no correlation between the hemolysis coefficients measured from the pooled plasma and the read counts for the 5 miRNAs selected for validation, indicating no effect of hemolysis on these miRNA expression levels. Further, the hemolysis coefficient did not correlate with the read count for the endogenous normalizer miR-28-3p. Hence, we proceeded to technical validation of the candidate miRNAs with real time-quantitative polymerase chain reaction (RT-qPCR). Analysis was performed using the same RNA used for small RNA-Seq.

#### 3.6.1. miR-9a-3p

The miR-9a-3p levels did not differ significantly between the sham-operated controls and naïve rats (1.7-fold, *p* > 0.05). The mTBI group had 8.8-fold higher miR-9a-3p levels than the sham-operated controls (*p* < 0.01) and 14.9-fold higher levels than the naïve rats (*p* < 0.05). The sTBI group also had higher miR-9a-3p levels than the mTBI group (3.2-fold, *p* < 0.05), sham-operated controls (28.6-fold, *p* < 0.01), and naïve rats (48.4-fold, *p* < 0.05) (Figure S4A).

#### 3.6.2. miR-136-3p

The miR-136-3p levels did not differ significantly between the sham-operated controls and naïve rats (2.8-fold, *p* > 0.05). The miR-136 = 3p levels of rats with mTBI did not differ from the sham-operated controls (1.1-fold, *p* > 0.05), but the miR-136-3p levels were 3.1-fold higher than those of naïve rats (*p* < 0.05). On the other hand, miR-136-3p levels in sTBI rats were higher than in the mTBI group (3.0-fold, *p* < 0.05), sham-operated controls (3.4-fold, *p* < 0.05), and naïve rats (9.5-fold, *p* < 0.05) (Figure S4B).

#### 3.6.3. miR-434-3p

The miR-434-3p levels were higher in the sham-operated controls than in naïve rats (3.3-fold, *p* < 0.05). The miR-434-3p levels did not differ between the mTBI group and sham-operated controls (0.9-fold, *p* > 0.05), but they were significantly higher in the mTBI group than in the naïve group (3.1-fold, *p* < 0.05). The miR-434-3p levels were higher in the sTBI group than in the mTBI group (3.2-fold, *p* < 0.01), sham-operated controls (3.0-fold, *p* < 0.01), and naïve rats (9.8-fold, *p* < 0.05) (Figure S4C).

The miR-153-3p and miR-15a-3p levels did not differ significantly between the naïve, sham, and mTBI groups (*p* > 0.05) (Figure S4D,E). Thus, the small RNA-Seq results for mTBI vs. naïve rats could not be validated for these 2 miRNAs, and therefore the sTBI samples were not analyzed for these miRNAs.

### 3.7. Validation of Regulated rno-miR-9a-3p, rno-miR-136-3p, and rno-miR-434-3p Plasma Levels in Whole Animal Cohort

Because small RNA-Seq was performed only on a subset of the animals, we evaluated the expression pattern of the technically validated miRNA candidates: miR-9a-3p, miR-136-3p, and miR-434-3p for the entire cohort. A separate 50-µL plasma aliquot from the EDTA-B tube for each animal was used in this step.

#### 3.7.1. Hemolysis

NanoDrop analysis revealed hemolysis in 10% (3/31) of the plasma aliquots. Hemolysis values measured from the plasma of the individual aliquots had a strong positive correlation (ρ = 0.919, *p* < 0.0001) with that measured from the EDTA-B tube immediately after centrifugation (prior to aliquoting).

#### 3.7.2. Plasma Levels of miR-9a-3p, miR-136-3p, and miR-434-3p Assessed with RT-qPCR

**Figure 6:**
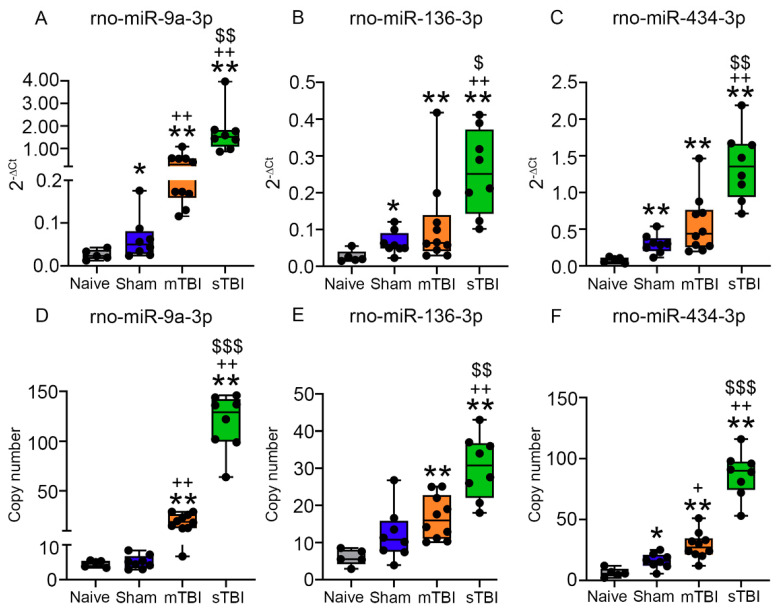
RT-qPCR and ddPCR confirmed the injury-severity-dependent increases in plasma miR-9a-3p, miR-136-3p, and miR-434-3p levels after experimental mTBI and sTBI. RT-qPCR indicated that (**A**) sham-operated controls had higher miR-9a-3p levels than naïve rats (*p* < 0.05). The mTBI group had higher miR-9a-3p levels than the naïve (*p* < 0.01) and sham-operated controls (*p* < 0.01). The sTBI group had higher miR-9a-3p levels than the naïve (*p* < 0.01), sham-operated controls (*p* < 0.01), and mTBI groups (*p* < 0.01). (**B**) The sham-operated controls had higher miR-136-3p levels than naïve animals (*p* < 0.05). The mTBI group had higher miR-136-3p levels than the naïve rats (*p* < 0.01), but similar levels to those in the sham-operated controls. The sTBI group had higher miR-136-3p levels than the naïve (*p* < 0.01), sham-operated controls (*p* < 0.01), and mTBI groups (*p* < 0.05). (**C**) Sham-operated controls also had higher miR-434-3p levels than the naïve (*p* < 0.01). The mTBI group had higher miR-434-3p levels only in comparison with naïve rats (*p* < 0.01). The sTBI group had higher miR-434-3p levels than the naïve rats (*p* < 0.01), sham-operated controls (*p* < 0.01), and mTBI groups (*p* < 0.01). (**D**–**F**) ddPCR analyses mostly revealed similar expression patterns as observed with RT-qPCR. The miR-9a-3p and miR-136-3p levels measured with ddPCR, however, did not differ between the naïve and sham groups. Moreover, with ddPCR, the mTBI group had increased miR-434-3p levels in comparison with the sham rats (*p* < 0.05). Abbreviations: Ct, cycle threshold; mTBI, mild traumatic brain injury; PCR, polymerase chain reaction; sham, sham-operated experimental controls; sTBI, severe traumatic brain injury. Statistical significances: * *p* < 0.05 and ** *p* < 0.01 compared to naïve, + *p* < 0.05 and ++ *p* < 0.01 compared to sham-operated controls, $ *p* < 0.05, $$ *p* < 0.01, and $$$ *p* < 0.001 compared to mTBI (Mann–Whitney *U* test).

The miR-9a-3p levels were higher in the sham-operated controls than in the naïve rats (2.4-fold, *p* < 0.05). The miR-9a-3p levels were higher in the mTBI group than in the sham (6.1-fold, *p* < 0.01) and naïve groups (14.8-fold, *p* < 0.01). The sTBI group had higher miR-9a-3p levels than all 3 of the other groups: mTBI (4.5-fold, *p* < 0.01), sham (27.2-fold, *p* < 0.01), and naïve (65.9-fold, *p* < 0.01) (Figure 6A).

The miR-136-3p levels were higher in the sham-operated controls than in the naïve animals (2.4-fold, *p* < 0.05). The miR-136-3p levels did not differ significantly between rats with mTBI and the sham group (1.8-fold, *p* > 0.05), but were higher in mTBI rats than in the naïve rats (4.3-fold, *p* < 0.01). The sTBI animals had higher miR-136-3p levels than all 3 of the other groups: mTBI (2.3-fold, *p* < 0.05), sham (4.0-fold, *p* < 0.01), and naïve (9.7-fold, *p* < 0.01) (Figure 6B).

The miR-434-3p levels were higher in the sham group than in the naïve rats (3.8-fold, *p* < 0.01). The miR-434-3p levels did not differ significantly between the mTBI group and the sham group (1.9-fold, *p* > 0.05), but were elevated in the mTBI group compared with naïve animals (7.1-fold, *p* < 0.01). The sTBI group had higher miR-434-3p levels than all 3 of the other groups: mTBI (2.4-fold, *p* < 0.01), sham (4.6-fold, *p* < 0.01), and naïve (17.2-fold, *p* < 0.01) (Figure 6C).

#### 3.7.3. Absolute Copy Numbers of miR-9a-3p, miR-136-3p, and miR-434-3p in Plasma Assessed with ddPCR

For miR-9a-3p, the sham-operated controls demonstrated similar mean copy numbers of miR-9a-3p compared with the naïve rats (1.1-fold, 5.1 vs. 4.6, *p* > 0.05). Mean copy numbers of miR-9a-3p in the mTBI group were 3.8-fold and 4.2-fold and higher compared to the sham (19.2 vs. 5.1, *p* < 0.01) and naïve groups (19.2 vs. 4.6, *p* < 0.01), respectively. The sTBI group demonstrated 6.2-fold, 23.5-fold, and 25.8-fold higher copy numbers of miR-9a-3p compared with the mTBI (118.8 vs. 19.2, *p* < 0.001), sham (118.8 vs. 5.1, *p* < 0.01), and naïve groups (118.8 vs. 4.6, *p* < 0.01), respectively (Figure 6D).

The mean copy numbers of miR-136-3p were similar between the sham-operated controls and naïve rats (2.0-fold, 12.2 vs. 6.0, *p* > 0.05). The mean copy numbers of miR-136-3p in the mTBI group did not differ from that in the sham group (1.4-fold, 16.7 vs. 12.2, *p* > 0.05), but were 2.8-fold higher than that in naïve rats (16.7 vs. 6.0, *p* < 0.01). Rats with sTBI had 1.8-fold, 2.5-fold, and 5.1-fold higher miR-136-3p levels than the mTBI (30.3 vs. 16.7, *p* < 0.01), sham (30.3 vs. 12.2, *p* < 0.01), and naïve groups (30.3 vs. 6.0, *p* < 0.01), respectively (Figure 6E).

Mean copy numbers of miR-434-3p were 2.6-fold higher in the sham group than in the naïve group (15.9 vs. 6.2, *p* < 0.05). The mTBI group had 1.8-fold and 4.6-fold higher miR-434-3p levels than the sham (28.7 vs. 15.9, *p* < 0.05) and naïve (28.7 vs. 6.2, *p* < 0.01) groups, respectively. The sTBI group had 3.0-fold higher miR-434-3p levels than the mTBI (87.0 vs. 28.7, *p* < 0.001), 5.5-fold higher than the sham group (87.0 vs. 15.9, *p* < 0.01), and 14-fold higher than the naïve group (87.0 vs. 6.2, *p* < 0.01) (Figure 6F).

Spearman’s analysis revealed a strong positive correlation between the RT-qPCR and ddPCR measurements for all three validated miRNAs: miR-9a-3p (ρ = 0.905, *p* < 0.0001), miR-136-3p (ρ = 0.625, *p* < 0.001), and miR-434-3p (ρ = 0.807, *p* < 0.0001).

**Figure 7:**
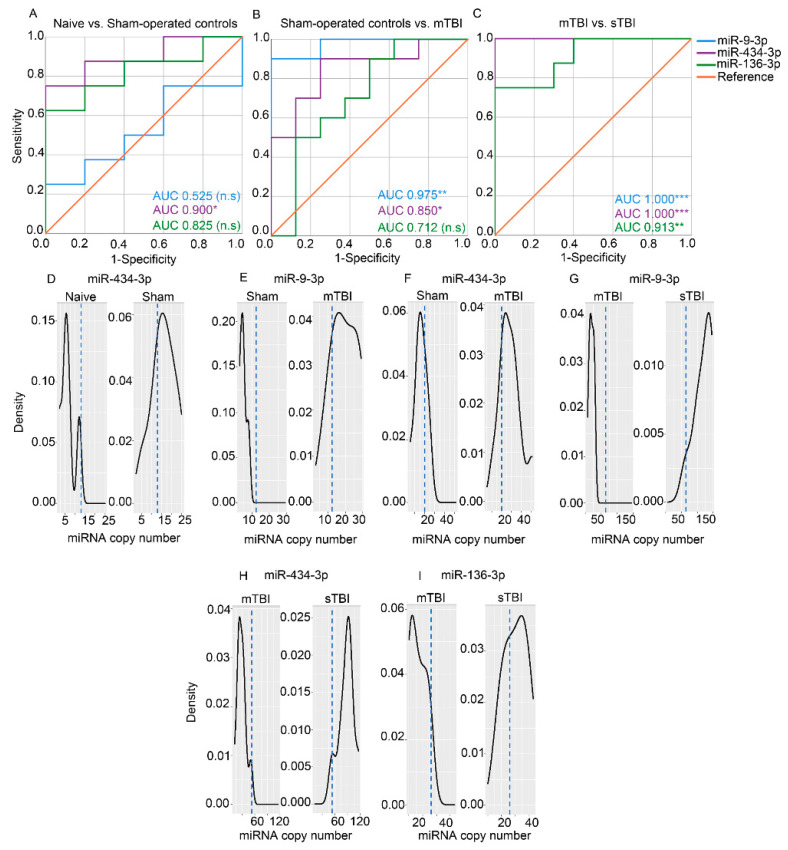
Receiver Operating Characteristic (ROC) and cut-off analysis indicated that plasma miRNAs separated the sham-operated controls from naïve rats (craniectomy effect), mTBI rats from sham-operated controls (TBI effect), and sTBI rats from mTBI rats (TBI severity effect). (**A**,**D**) Plasma miR-434-3p levels separated the sham-operated controls from the naïve rats with 75% sensitivity and 100% specificity (cut-off 13 copies, AUC 0.900, *p* < 0.05), whereas plasma miR-9a-3p and miR-136-3p did not separate the groups. (**B**,**E**,**F**) Plasma miR-9a-3p levels separated the mTBI group from the sham-operated controls with 90% sensitivity and 100% specificity (cut-off 12 copies, AUC 0.975, *p* < 0.01) and miR-434-3p levels with 90% sensitivity and 75% specificity (cut-off 20 copies, AUC 0.850, *p* < 0.05). miR-136-3p levels were similar between the two groups. (**C**,**G**–**I**) Plasma miR-9a-3p levels separated the sTBI group from the mTBI group with 100% sensitivity and 100% specificity (cut-off 64 copies, AUC 1.000, *p* < 0.001), miR-434-3p with 100% sensitivity and 100% specificity (cut-off 53 copies, AUC 1.000, *p* < 0.001) and miR-136-3p 75% sensitivity and 100% specificity (cut-off 26 copies, AUC 0.913, *p* < 0.05). Abbreviations: AUC, area under the curve; miRNA, microRNA; mTBI, mild traumatic brain injury; n.s, not significant; sham, sham-operated experimental controls; sTBI, severe traumatic brain injury. Statistical significances: * *p* < 0.05, ** *p* < 0.01, *** *p* < 0.001 (Mann-Whitney *U* test).

Receiver operating characteristic analysis. The AUC for pairwise group comparisons of the absolute copy numbers assessed with ddPCR are summarized in Supplementary Table S1. The receiver operating characteristic (ROC) curves and the cut-off plasma miRNA copy numbers for miR-9a-3p, miR-136-3p, and miR-434-3p distinguishing the sham-operated controls from the naïve animals (indicating an effect of craniectomy on plasma miRNA levels without a TBI impact), the rats with mTBI from the sham-operated controls (indicating a mild impact effect), and the rats with sTBI from the mTBI (indicating an effect of the impact severity) are shown in Figure 7.

The miR-9a-3p copy number did not separate sham-operated controls from naïve rats (AUC 0.525, *p* > 0.05). It did, however, separate the mTBI group from the sham-operated controls (cut-off of copy number 12, 90% sensitivity and 100% specificity) and from naïve rats (cut-off of copy number 7, 100% sensitivity and 100% specificity). A plasma miR-9a-3p copy number of 64 separated the sTBI group from the mTBI group, sham-operated controls, and naïve rats (100% sensitivity and 100% specificity for all).

The miR-136-3p copy number did not distinguish sham-operated controls from naïve rats (AUC 0.825, *p* > 0.05) or the mTBI group from the sham-operated control group (AUC 0.712, *p* > 0.05). The miR-136-3p copy number separated the mTBI group from the naïve group (cut-off of copy number 10, 100% sensitivity and 100% specificity). Furthermore, miR-136-3p copy number separated the sTBI group from the mTBI group (cut-off of copy number 26, 75% sensitivity and 100% specificity), from sham-operated controls (cut-off of copy number 18, 100% sensitivity and 88% specificity), and from naïve rats (cut-off of copy number 18, 100% sensitivity and 100% specificity).

A plasma miR-434-3p copy number of 13 distinguished the sham-operated controls from naïve rats (75% sensitivity and 100% specificity). Furthermore, the miR-434-3p copy number separated the mTBI group from the sham-operated controls (cut-off of copy number 20, 90% sensitivity and 75% specificity) and naïve rats (cut-off of copy number 12, 100% sensitivity and 100% specificity). A plasma miR-434-3p copy number of 53 separated the sTBI group from the mTBI group, sham-operated controls, and naïve rats (100% sensitivity and 100% specificity for all).

### 3.8. ddPCR of hsa-miR-9-3p and hsa-miR-136-3p in Human TBI Plasma

We then assessed if the changes in miRNA levels detected in the animal model could be found in humans with TBI. Of the three miRNAs analyzed first with RT-qPCR, miR-434-3p was not detected in human samples. Therefore, only hsa-miR-9-3p and hsa-miR-136-3p were analyzed with ddPCR in human plasma samples.

#### 3.8.1. Hemolysis

Hemolysis was observed in 21% (3/14) of the control samples. Among the mTBI plasma samples, 20% (3/15) were hemolyzed. Neither one of the two sTBI plasma samples were hemolyzed.

#### 3.8.2. Effect of Sex, Age, and Injury-Sampling Interval on Plasma miR-9-3p and miR-136 Levels

No sex differences were observed in miR-9-3p and miR-136-3p levels in the mTBI or control cases. Further, miR-9-3p and miR-136-3p levels measured from the mTBI and control cases did not correlate with age, and those measured in mTBI cases did not correlate with hours to sampling postinjury.

#### 3.8.3. Absolute Copy Numbers for miR-9-3p and miR-136-3p in Plasma Assessed with ddPCR

From unnormalized RNA. When analyzed from the unnormalized RNA, the mean miR-9-3p copy number in the mTBI group was 1.8-fold than that in controls (57 vs. 31, *p* > 0.05). Further analysis indicated that 4 of the 15 mTBI patients had miR-9-3p levels higher than one standard deviation (SD) above the control (data points marked with diamonds in the mTBI group, Figure S5A). In the 2 sTBI cases the mean miR-9-3p levels were 5.3-fold than that in the mTBI group (302 vs. 57) and 9.7-fold than that in the control group (302 vs. 31).

The miR-136-3p level in the mTBI group was 2.3-fold than that in controls (859 vs. 380, *p* > 0.05). Of the 15 mTBI patients, 3 had miR-136-3p levels higher than 1 SD above the control mean. These three patients also had elevated miR-9-3p levels (higher than control mean + 1SD, data points marked with diamonds in the mTBI group, Figure S5B). The miR-136-3p level in the sTBI patients were similar to that in the mTBI or control groups (Figure S5B).

From small RNA-concentration normalized RNA. Small RNA concentration measurements from all TBI and control plasma samples indicated that eluted total small RNA concentrations across samples ranged from 0.18 to 1.21 ng/µL (mean 0.4 ng/µL, percentage of coefficient of variation (CV%) 55%). To reduce the bias in the estimated miRNA copy numbers as a result of this variation in eluted total small RNA concentration, we normalized the small RNA concentration. With this normalization, similar small RNA concentrations are used as input for the reverse transcription reaction. With the normalization, the within group sample-to-sample variability was reduced for both miR-9-3p and miR-136-3p. The CV% for miR-9-3p in the unnormalized control and mTBI groups was 81% and 93%, respectively. In the normalized samples, the CV% decreased to 58% and 60%, respectively. Similarly, the CV% for miR-136-3p in the unnormalized control and mTBI groups was 92% and 231%, respectively, whereas in the normalized samples, the CV% decreased to 91% and 109%, respectively. For the 2 sTBI patients, the within group sample-to-sample variability was reduced in the small RNA concentration-normalized samples compared with the unnormalized samples for miR-9-3p (57% vs. 78%), but not for miR-136-3p (86% vs. 71%).

**Figure 8:**
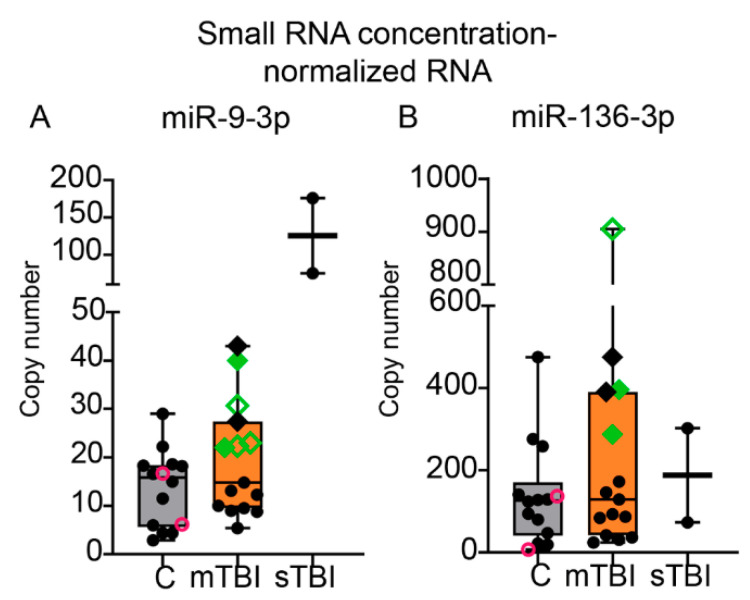
ddPCR revealed elevated miR-9-3p and miR-136-3p levels in a subpopulation of mTBI patients that also had high plasma S100B levels. Whisker-plots (box extends from the 25th–75th percentiles) shows that (**A**) mean miR-9-3p copy numbers (per 20 µL of PCR reaction volume, normalized for total small-RNA concentration, *y*-axis) were similar between the mTBI and controls (*x*-axis, *p* > 0.05). In 7 of the 15 mTBI patients (diamonds), however, miR-9-3p levels were higher than mean + 1SD of control mean (>21 copies). miR-9-3p levels in the sTBI patients were 9.2-fold compared to that in the controls and 6.5-fold compared to that in the mTBI group, suggesting a trend toward an injury-severity effect. (**B**) Mean miR-136-3p copy numbers did not differ between the mTBI patients and controls (*p* > 0.05). In 5 of the 15 mTBI patients (diamonds), miR-136-3p copy number was higher than mean + 1SD of the control mean (>264 copies). Unlike miR-9-3p, data from the sTBI patients did not suggest any injury-severity effect. In two mTBI patients, the miR-9-3p and miR-136-3p levels were higher than mean + 1SD of that in controls, and the plasma S100B levels were higher than the reference of 0.1 µg/L (solid green diamonds). Statistical test: (Mann–Whitney *U* test). Abbreviations: C, age-matched controls; mTBI, mild traumatic brain injury; sTBI, severe traumatic brain injury. Symbol key: open pink circles indicate the controls age-matched with the sTBI patients; black solid diamonds indicate the mTBI patients with plasma S100B level < 0.1 µg/L but plasma miR-9-3p and miR-136-3p levels > mean + 1SD of the controls; green solid diamonds indicate the mTBI patients with plasma S100B level > 0.1 µg/L as well as plasma miR-9-3p and miR-136-3p levels > mean + 1SD of the controls; open green diamonds indicate the mTBI patients with plasma S100B level >0.1 µg/L and either plasma miR-9-3p level or miR-136-3p level > mean + 1SD of the controls.

With small RNA concentration-normalization, the pattern of miR-9-3p or miR-136-3p expression between the mTBI and control groups remained similar to that observed in the analysis from unnormalized RNA. The mean miR-9-3p copy number in the mTBI group was 1.4-fold than that in controls (19 vs. 14, *p* > 0.05). With the normalization, 7 of the 15 mTBI patients had miR-9-3p levels higher than 1 SD above the control mean (data points marked with diamonds in the mTBI group, Figure 8A). In the sTBI group, the miR-9-3p levels were 6.5-fold as compared to that in the mTBI group (126 vs. 20) and 9.2-fold than that in the control group (126 vs. 14).

The miR-136-3p level in the mTBI group was 1.6-fold than that in controls (220 vs. 138, *p* > 0.05). With the normalization, 5 of the 15 patients had miR-136-3p levels higher than 1SD above the control mean (data points marked with diamonds in the mTBI group, Figure 8B). Of these 5 patients, 4 also had elevated miR-9-3p levels (higher than control mean + 1SD, data points marked with black and green solid diamonds in the mTBI group, Figure 8B). The miR-136-3p level in the sTBI group was 0.9-fold (188 vs. 220) and 1.4-fold (188 vs. 138) compared to that in the mTBI and control groups, respectively.

ROC analysis. ROC analysis for miR-9-3p and miR-136 levels measured with ddPCR from unnormalized RNA did not distinguish the controls from the mTBI patients (miR-9-3p: AUC 0.657, *p* > 0.05; miR-136-3p: AUC 0.476, *p* > 0.05). Using small RNA concentration normalized RNA produced similar results (miR-9-3p: AUC 0.638, *p* > 0.05, miR-136-3p: AUC 0.590, *p* > 0.05) (Figure S6).

#### 3.8.4. Association of miR-9-3p and miR-136 Levels with Plasma S100B Concentrations

Among the 15 mTBI patients, 11 demonstrated elevated S100B levels (>0.1 µg/L [12]). No correlations were observed between miR-9-3p or miR-136-3p and S100B levels measured from the mTBI cases. Five of the seven mTBI patients with normalized plasma miR-9-3p levels higher than1 SD above the control mean also had elevated S100B levels (data points marked as green open and solid diamonds in the mTBI group, Figure 8A). Similarly, three of the five mTBI patients with normalized plasma miR-136-3p levels higher than 1 SD above the control mean also had elevated S100B levels (data points marked as green open and solid diamonds in the mTBI group, Figure 8B).

## 4. Discussion

The present study evaluated whether mTBI leads to altered plasma miRNA levels and whether some of these miRNAs could serve as diagnostic biomarkers for experimental and/or clinical mTBI and produced four main findings. First, sequencing plasma miRNAs at 2 days postinjury revealed that mTBI led to an upregulation of 15 plasma miRNAs compared with naïve rats. We validated the increases of three miRNAs: miR-9a-3p, miR-136-3p, and miR-434-3p. Second, all three of these miRNAs exhibited an impact-severity-dependent increase in our experimental lateral FPI model in rats, being higher after sTBI than mTBI. Third, even sham surgery, including craniectomy without lateral FPI impact, altered the plasma miRNA profile compared with that in naïve rats. Fourth, the increase in miR-9a-3p and miR-136-3p levels was also detected in a subgroup of patients with mTBI.

### 4.1. mTBI Led to an Acute Increase in Plasma miR-9a-3p, miR-136-3p, and miR-434-3p Levels

The miRNAs identified from plasma sequencing of the rats at 2 days after experimental mTBI were sorted into descending order by the normalized mean absolute coefficient values from logistic regression CV. The coefficient mass aggregated on a set of eight miRNAs, from which we selected the top five miRNAs for independent validation. Among these, three miRNAs were also significantly regulated in the DE analysis. The plasma levels of these three miRNAs, miR-9a-3p, miR-136-3p, and miR-434-3p, survived the independent validation by both RT-qPCR and ddPCR when compared between the mTBI and naïve rats.

What pathologies do the altered plasma miRNA levels represent? Our study revealed that increased plasma miR-9a-3p levels can distinguish mTBI rats at 2 days post-TBI from naïve rats with excellent diagnostic accuracy. We also found that plasma miR-9-3p levels were increased in a subpopulation of patients with mTBI, some of whom also had increased plasma S100B levels, which is a more established diagnostic biomarker for mTBI [12,13]. MiR-9 is one of the most conserved and abundant miRNAs in the vertebrate central nervous system (CNS) [45,46,47]. Expression of miR-9 is detected in neuronal progenitors in the embryonic and developing brain, and in mature postmitotic neurons in adults [48]. A major function of miR-9 is to regulate neuronal progenitor proliferation in the developing brain [48]. Specifically, miR-9-3p contributes to synaptic plasticity and memory formation in adult mouse brains [49]. Aberrant expression of miR-9 is observed in neurodegenerative diseases, as well as in brain cancers [48,49,50]. Some studies have also reported miR-9 expression in normal peripheral tissues [51,52,53]. miR-9 acts as both an oncogene and a tumor suppressor, with aberrant expression levels detected in malignant transformations outside the CNS [48,54]. Importantly, its level in normal plasma is very low, as indicated by a low copy number in naïve rats and also in the human controls in our study. Similar to our observation, another recent study identified increased miR-9a-3p levels in plasma exosomes of rats with weight-drop–induced TBI compared with sham-operated controls [55]. Elevated serum miR-9 levels were also reported in a group of American-style football players with at least six years of playing experience and 41% with previously reported concussion, in comparison with nonathlete controls [24]. Altered miR-9 levels are also reported in synergy with another brain-enriched miRNA, miR-124, in the serum and CSF of patients with acute ischemic stroke [56,57,58]. Taken together, these data suggest that the increased plasma miR-9-3p levels observed in the present study after mTBI in rats and humans indicate neuronal dysfunction.

The miRNA miR-136-3p is a novel plasma biomarker candidate of mTBI. In the present study, we observed that a 3-fold increase in the plasma miR-136-3p level in rats with mTBI distinguished them from the naïve rats with 100% sensitivity and specificity. Importantly, our study is the first to report the presence of miR-136 in human plasma after TBI. miR-136-3p is enriched in primary cultured neurons from postnatal day 1 rat cortex [59]. Upregulation of miR-136-3p expression was reported in synaptoneurosomes at the preclinical stage of prion disease [60]. In contrast, downregulation of miR-136-3p expression is observed in glioma tissue of patients with a more aggressive and/or poor prognostic phenotype [61,62]. miR-136 exerts a protective function on neurons in a model of spinal cord ischemia injury [63]. miR-136-3p expression is also detected outside the CNS [64,65,66]. Circulating and CSF miR-136-3p levels are candidate biomarkers for both Parkinson’s disease and Alzheimer’s disease [67,68,69,70]. In patients with amyotrophic lateral sclerosis, serum miR-136-3p levels correlate with rapid disease progression [71]. Selvamani et al. reported that miR-136 levels are almost 2-fold higher after cerebral ischemia induced with middle cerebral artery occlusion, particularly in ischemic adult female rats compared with male rats [72], suggesting the potential role of this miRNA in regulating stroke severity sex-dependently. No previous reports, however, have linked miR-136 to TBI. Our study reports excellent diagnostic accuracy of miR-136-3p in experimental mTBI. Although we did not find similar diagnostic accuracy for miR-136 in human mTBI, we found elevated plasma miR-136 levels in a subpopulation of mTBI patients. The difference between the experimental and clinical data probably relates to the greater heterogeneity of human mTBI compared with experimental mTBI. Taken together, these promising data on miR-136 as a potential diagnostic biomarker for mTBI warrant further studies on a larger population of mTBI patients.

miR-434-3p is another novel plasma miRNA biomarker candidate of mTBI identified in our analysis. Increased plasma miR-434-3p levels at 2 days post-TBI had 100% sensitivity and specificity as a diagnostic biomarker for experimental mTBI after lateral FPI compared with naïve rats. miR-434-3p is also enriched in neurons [59]. Analysis from primary neuronal cultures further indicates that miR-434-3p can regulate the neuronal transcriptome during stress conditions [73]. A 2-fold increase in circulating miR-434-3p levels was identified as a biomarker for epileptogenesis in a pilocarpine-induced mouse model of status epilepticus [74]. In addition to the CNS, miR-434 expression is also reported in peripheral tissues, such as the rat skeletal muscle and atherosclerotic aorta [75,76,77]. Circulating miR-434-3p is a candidate biomarker of muscle aging and damage [78,79], as well as of Gram-positive bacterial infection [80]. Serum miR-434-3p levels are increased in a mouse weight-drop model of mTBI [31]. miR-434 is a rodent-specific miRNA [81], undetected in humans. Thus, miR-434-3p levels in human plasma were not detected and its biomarker potential for clinical TBI could not be explored. Nevertheless, identification of preclinical biomarkers for mTBI is critical for advancing progress in treatment discovery and development.

In addition to miR-9a-3p, miR-136-3p, and miR-434-3p, 2 other miRNA candidates were identified by the logistic regression analysis as important features distinguishing the mTBI group from naïve rats: miR-153-3p and miR-15a-3p. The RT-qPCR analysis, however, did not successfully validate these candidates. A plausible reason for this is the low abundance of the miRNAs in plasma as indicated by a normalized count per million < 10 in the small RNA-Seq data. These findings indicate that miRNAs with a low expression abundance identified from machine-learning or DE-based analysis of small RNA-Seq should be treated with caution, and PCR-based independent validation of such candidates is critical for evaluating their biomarker potential.

To summarize, our pipeline for miRNA diagnostic biomarker discovery in the lateral FPI model of TBI identified plasma miR-9a-3p, miR-136-3p, and miR-434-3p as candidate diagnostic biomarkers for mTBI. Further, acute plasma miR-9-3p and miR-136-3p levels were also increased in a subpopulation of patients with mTBI, some of whom also exhibited increased plasma S100B levels.

### 4.2. An Impact Severity -Dependent Increase was Observed in the Plasma miRNA Signature

Previous studies in rats with lateral FPI demonstrated that an impact pressure of 1.5 atm successfully recapitulated the clinical findings of mTBI [82,83,84]. In correlation with previous studies, our mTBI cohort exhibited no postimpact mortality, whereas 20% acute postimpact mortality was observed in the sTBI cohort. Further, the duration of postimpact apnea was also significantly lower in the mTBI cohort in comparison to the sTBI. Nissl-stained brain sections from the mTBI rats at 2 days post-TBI revealed some extent of neuronal loss at the lesion core, but the perilesional haemorrhage and neuronal loss was more prominent in the sTBI cases. In addition, the the miRNAs investigated in detail showed a clear injury-severity-dependency. In particular, the levels of these miRNAs were low in normal plasma and increased according to the severity of the impact force in the following order: naïve < craniectomized experimental controls < mTBI < sTBI.

Several recent reports have questioned the use of craniectomized animals as controls for TBI, as the craniectomy itself induces significant morphologic damage and behavioral abnormalities [85,86,87,88,89,90,91]. For example, a study reported differences in glial fibrillary acidic protein and ubiquitin *C*-terminal hydrolase L-1 levels at 4 h and 24 h postsurgery between the sham-operated experimental controls in different TBI models, including FPI, penetrating ballisticlike brain injury and controlled cortical impact [86]. Further, altered trauma markers have been detected in the brain tissue of craniectomized sham-operated controls [87,88,90]. Gait impairment post-TBI is also attributed in part to the effect of craniectomy [89]. In our study, small RNA seq analysis revealed 41 differentially expressed miRNAs in the plasma of sham-operated control rats at 2 days postsurgery compared with the naïve rats, whereas only four differentially expressed miRNAs were identified when the sham group was compared to the mTBI group. In addition, levels of the three PCR-validated miRNA biomarker candidates tended to be higher in the sham group than in the naïve rats. Thus, our findings strengthened the view that even a craniectomy with the dura kept intact can affect the plasma levels of presumably brain-derived miRNAs and this should be taken into consideration in experimental biomarker studies. It also underscores the importance of including naïve animals in the study design along with the craniectomized sham-operated controls. Further, it necessitates comparison of the findings from our current study with that of the experimental TBI models that do not involve craniectomy, such as the weight-drop (without craniectomy) or the blast TBI [92].

The rats receiving sTBI had higher plasma levels of all the 3 validated miRNA candidates compared with the mTBI group, revealing an impact severity-dependent increase. To the best of our knowledge, only one previous preclinical study using a mouse weight-drop model of mTBI investigated alterations in serum miRNA levels in response to variations in TBI injury severity [31]. Sharma et al. reported increased serum miR-434-3p levels with the increase in impact severity [31]. In addition to miR-434-3p, we confirmed that plasma miR-9a-3p and miR-136-3p levels increased in correspondence with an increase in the injury severity in our rat model of lateral FPI. Taken together, our findings indicate that increasing the hit pressure leads to a more severe brain injury, along with an impact severity-dependent increase in plasma levels of specific brain-enriched miRNAs that act as a noninvasive signal for the injury severity.

In our analysis of human plasma samples, the brain-enriched miR-9-3p levels were 6.5-fold in the patients with sTBI in comparison to the mTBI, whereas miR-136-3p levels were only 0.9-fold. This suggests that in the patient samples miR-9-3p performs better in predicting the injury severity than miR-136-3p but needs to be confirmed in a larger patient cohort. Further, when comparing patients with mTBI to the controls, fold changes in plasma miR-9-3p and miR-136-3p levels were smaller than that observed in the rats with mTBI in comparison to the naïve in the lateral FPI model. This suggests that the inherent biological heterogeneity in humans can play a critical role in determining plasma miRNA levels, thereby requiring validation in a larger patient population. It is important to note that the control population in our study had no evident brain injuries reported, but some had complaints of headaches, arthrosis related pain etc. It remains to be investigated if the plasma miRNA levels in the control population are affected by these factors.

Both miR-9-3p and miR-136-3p have been identified to be brain-enriched in humans [93]. On the other hand, the S100B protein is expressed in muscle and adipose tissue and often reported to be elevated in patients with musculoskeletal injury without a brain injury [15]. Thus, miR-9-3p and miR-136-3p may be more sensitive and specific biomarkers of mTBI and of injury severity in comparison to S100B.

## 5. Conclusions

We identified increased plasma levels of three brain enriched miRNAs (miR-9a-3p, miR-136-3p, and miR-434-3p) at 2 days after experimental mTBI that distinguished injured rats from naïve rats with excellent diagnostic accuracy. In addition, we identified that a subpopulation of patients with mTBI exhibit increased plasma miR-9-3p and miR-136-3p levels in conjunction with increased plasma S100B levels. Further, our data reveals that the plasma miRNA levels correlate with the severity of brain injury in the lateral FPI model. A similar trend of correlation was observed for miR-9-3p in the patient population, indicating its potential to be translated in the clinic. Importantly, plasma miRNA levels were also altered in the sham surgery group in comparison with the naïve rats, indicating that even a craniectomy with the dura kept intact can induce alterations in the plasma miRNA profile. Thus, naïve rats should be included as experimental study controls in TBI biomarker studies. Overall, miR-9-3p and miR-136-3p are novel biomarker candidates that should be further developed for the detection of mTBI in experimental models and humans.

Study Limitations. At the time of conducting this study, plasma samples were available for only 15 mTBI and 2 sTBI patients from the Kuopio University hospital. Thus, our findings regarding the plasma miR-9a-3p and miR-136-3p levels in the clinical TBI samples are preliminary and require further evaluation in a larger patient population.

## Figures and Tables

**Table 1:** Clinical characteristics of the human patients with mild and severe traumatic brain injury, and controls.

Clinical Characteristics	Controls (*n* = 14)	mTBI (*n* = 15)	sTBI (*n* = 2)
Age (mean ± SD)	63 ± 6	68 ± 12	63 ± 3
Female (*n* (%))	4 (29)	5 (33)	0 (0)
GCS (mean ± SD)	-	14.8 ± 0.6	3.5 ± 0.7
Post-traumatic amnesia (*n* (%))	-	12 (80)	-
Hours from injury to plasma sampling (mean ± SD)	-	11 ± 12	-
Plasma S100B (µg/L) (mean ± SD)	-	0.2 ± 0.1	-
Plasma S100B > 0.1 µg/L (*n* (%))	-	11 (73)	-
Alcohol consumed (*n* (%))	-	3 (20)	1 (50)

Abbreviations: GCS, Glasgow coma scale; mTBI, patients with mild traumatic brain injury; *n*, number of cases; SD, standard deviation; sTBI, patients with severe traumatic brain injury.

## Data Availability

The small RNA sequencing data has been deposited to the Gene Expression Omnibus (GEO) under accession No. GSE159011. The processed sequencing data and validation results from RT-qPCR and ddPCR presented in this study are available on request from the corresponding author.

## Supplementary Materials

The following are available online at https://www.mdpi.com/1422-0067/22/4/1563/s1.
