# Supplementary material for: Plasma miR-9-3p and miR-136-3p as Potential Novel Diagnostic Biomarkers for Experimental and Human Mild Traumatic Brain Injury
Source: Int J Mol Sci. 2021 Feb 4;22(4):1563. doi: 10.3390/ijms22041563 (PMC7913923; doi:10.3390/ijms22041563)
Supplement: Supplementary file 1 [file ijms-22-01563-s001.pdf]

Supplementary Data

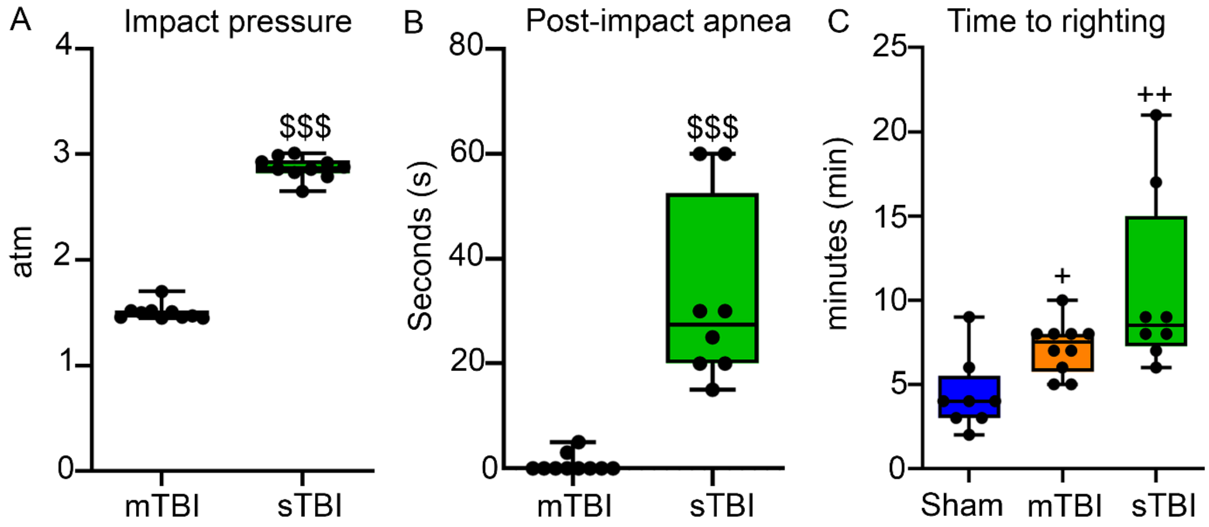

Supplementary Figure S1. Whisker boxplots showing impact pressure, duration of post-impact apnea, and time to righting. (A) Impact pressure was 1.9-fold higher in the sTBI group compared to mTBI ($p<0.001$). **(B)** The duration of post-impact apnea was also higher in the sTBI group compared to mTBI ($p<0.001$). **(C)** Righting reflex was suppressed in both mTBI ($p<0.05$) and sTBI groups ($p<0.01$) compared with the sham-operated controls. Statistical significance: +$p<0.05$; ++$p<0.01$ (compared with sham-operated controls); $$$$p<0.001$ (compared with mTBI) (Mann-Whitney's *U* test). Abbreviations: atm, standard atmosphere; mTBI, mild traumatic brain injury; sham, sham-operated experimental controls; sTBI, severe traumatic brain injury (Mann-Whitney's *U* test).

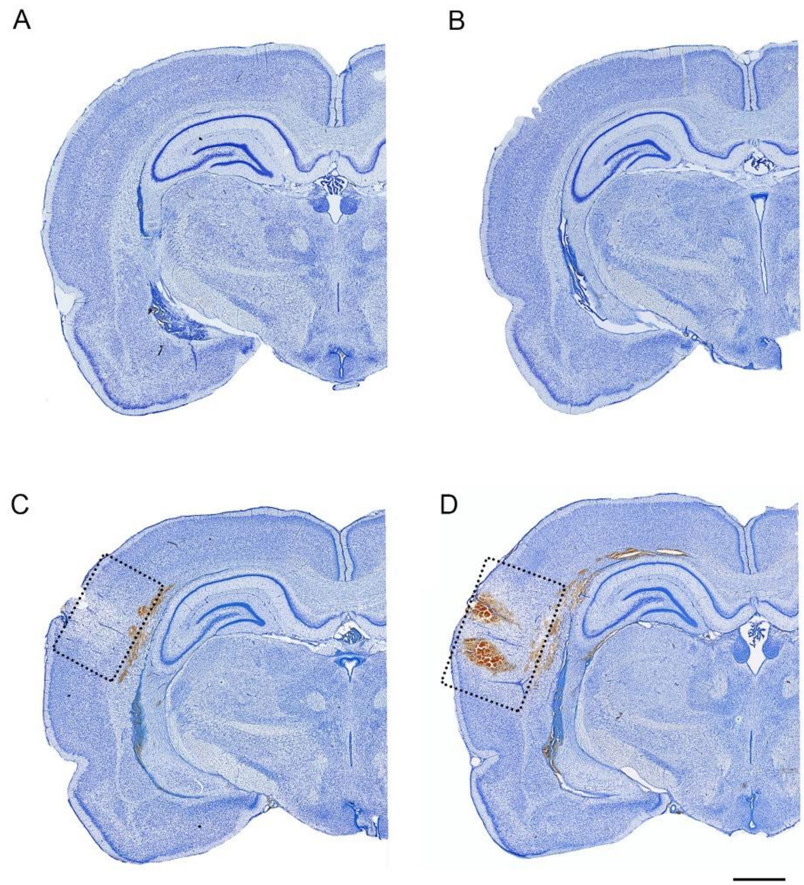

Supplementary Figure S2. Brightfield photomicrographs of representative Nissl-stained coronal brain sections from the rostro-caudal level corresponding to the lesion epicenter of rats killed at 2 d after operation. (A) A naïve rat. **(B)** Sham-operated experimental control with craniectomy. **(C)** A rat with a mild traumatic brain injury (mTBI). Note neuronal cell loss at the cortical lesion core (marked with a dashed box) and hemorrhage in the deep layers of the cortex and external capsule at 2 d post-TBI. **(D)** A rat with severe TBI (sTBI). Note that cortical neuronal loss and hemorrhage in the external capsule were more prominent than that after mTBI (panel C). Scale bar =1 mm

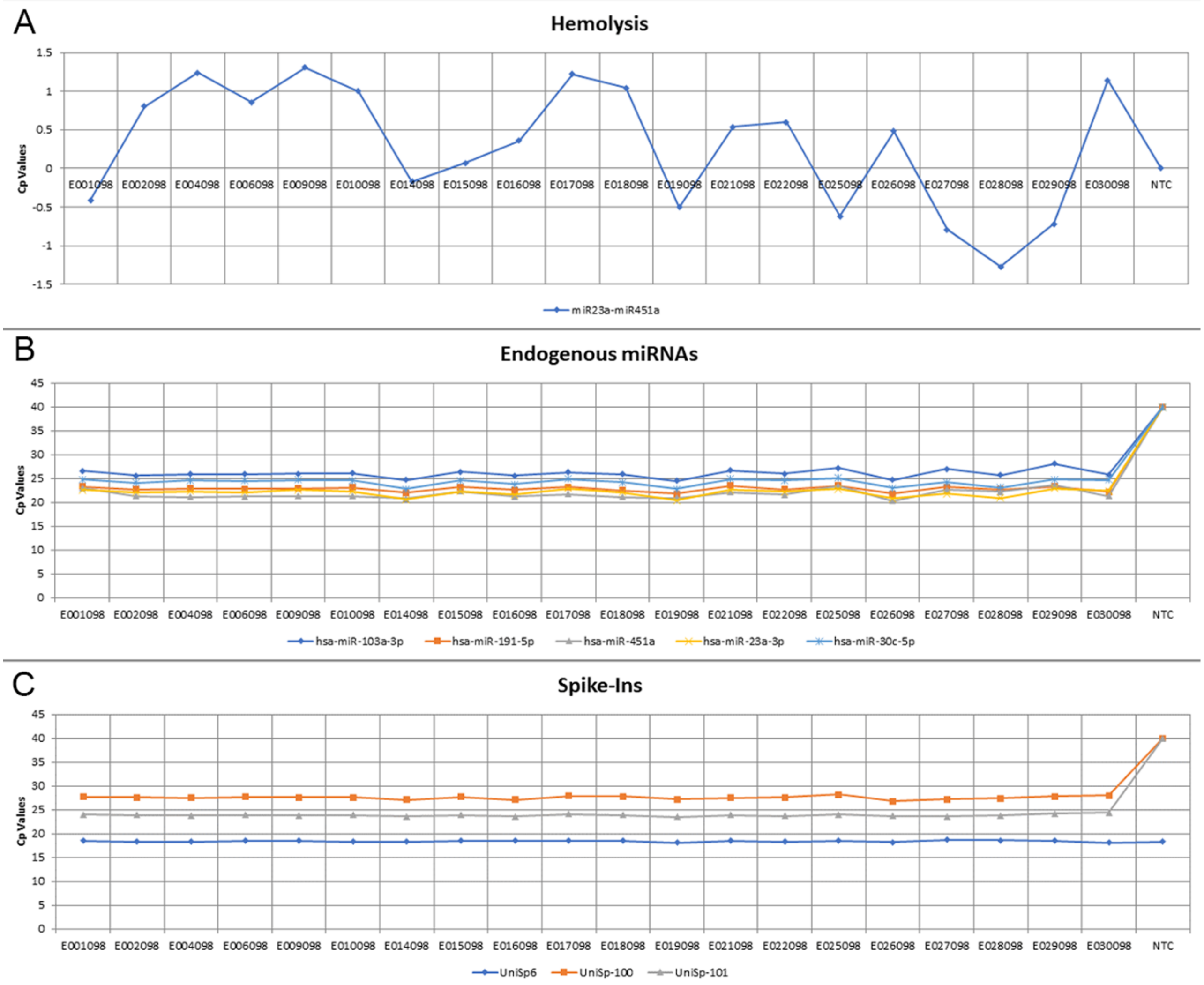

Supplementary Figure S3. Quality control of plasma samples and extracted RNA prior to library preparation and sequencing by Qiagen. (A) Presence of hemolysis in 20 plasma samples (E codes) and 1 negative control (NTC) used for RNA extraction was measured with the ΔCq (miR-23a - miR-451) method. ΔCq>5 was considered abnormal. **(B)** To confirm the success of RNA extraction, levels of 5 abundantly expressed endogenous plasma miRNAs were quantified: hsa-miR-103a-3p, hsa-miR-191-5p, hsa-miR-451a, hsa-miR-23a-3p, and hsa-miR-30c-5p. **(C)** Quality of inhibition of enzymatic reactions during RNA extraction was measured by analyzing the expression of synthetic spike-in controls. Abbreviations: Cp, crossing point.

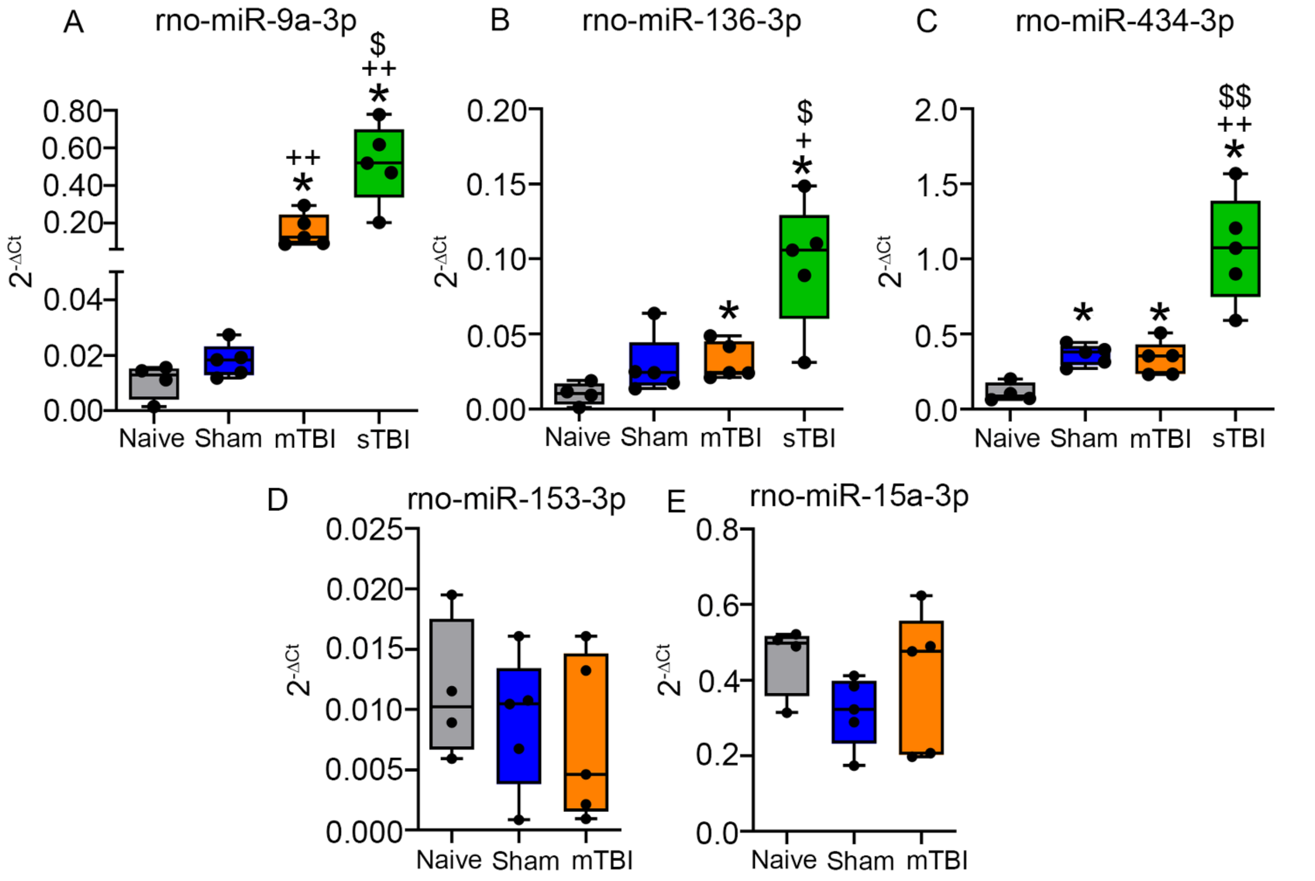

Supplementary Figure S4. RT-qPCR based technical validation of miR-seq data. (A) The naïve and sham-operated controls revealed similar miR-9a-3p levels. The mTBI group had higher miR-9a-3p levels than both naïve rats ($p<0.05$) and sham-operated controls ($p<0.01$). The sTBI group had higher miR-9a-3p levels than the naïve group ($p<0.05$), sham-operated controls ($p<0.01$), and the mTBI group ($p<0.05$). **(B)** The naïve and sham-operated controls had similar miR-136-3p levels. The mTBI group had higher miR-136-3p levels than naïve rats ($p<0.05$), but comparable to those in sham-operated controls ($p>0.05$). The sTBI group had elevated miR-136-3p levels compared with the naïve ($p<0.05$), sham-operated controls ($p<0.05$), and mTBI groups ($p<0.05$). **(C)** Sham-operated controls had higher miR-434-3p levels than the naïve animal ($p<0.05$). Similarly, the mTBI group had higher miR-434-3p levels compared with naïve rats ($p<0.05$), but similar levels in comparison to the sham-operated experimental controls. The sTBI group had higher miR-434-3p levels compared with the naïve rats ($p<0.05$), sham-operated controls ($p<0.01$), and mTBI groups ($p<0.01$). **(D-E)** No differences in miR-153-3p and miR-15a-3p levels were observed between the naïve, sham, and mTBI groups. Abbreviations: Ct, cycle threshold; mTBI, mild traumatic brain injury; sham, sham-operated controls; sTBI, severe traumatic brain injury. Statistical significance: *$p<0.05$ compared to naïve, +$p<0.05$ and ++$p<0.01$ compared to sham-operated controls, \$$p<0.05$ and \$\$$p<0.01$ compared to mTBI (Mann-Whitney's *U* test).

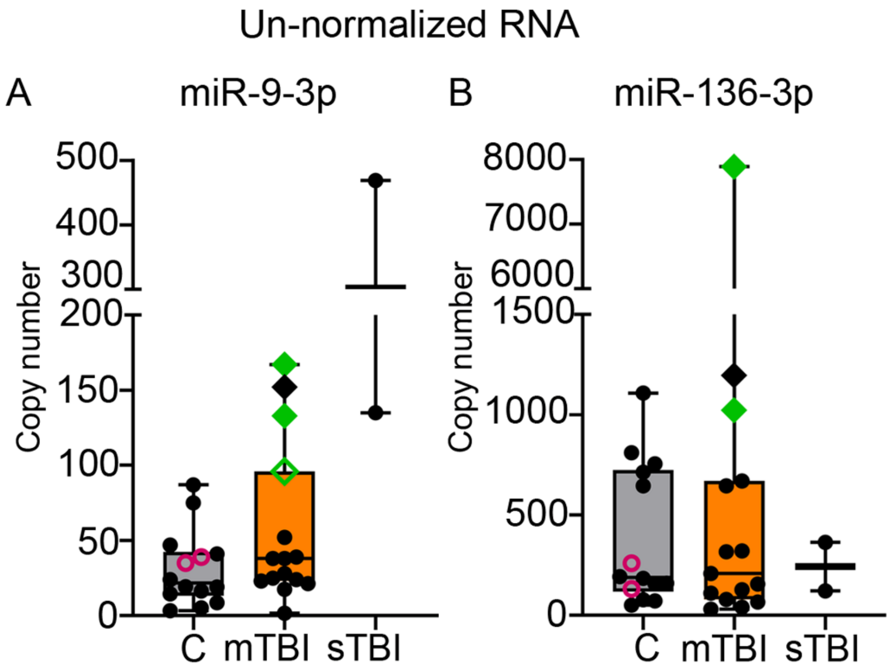

Supplementary Figure S5. A subpopulation of human mTBI patients had plasma miR-9-3p and miR-136-3p levels higher than 1 SD above the mean of that in controls as well as elevated plasma S100B levels. Whisker box-plots (box extends from the 25th -75th percentiles) show that **(A)** mean miR-9-3p copy numbers (per 20 µl of PCR reaction volume, measured from un-normalized RNA, y-axis) were similar between the mTBI and the controls ($p>0.05$). Interestingly, 4 of 15 mTBI patients (diamonds) had miR-9-3p number higher than mean+1SD of control mean (>56 copies). miR-9-3p levels in the sTBI patients was 9.7-fold than that of controls and 5.3-fold than that of the mTBI group. **(B)** Mean miR-136-3p copy numbers did not differ between the mTBI patients and controls ($p>0.05$). In 3 of 15 mTBI patients (diamonds), the miR-136-3p copy number was higher than the mean+1SD of the control mean (>729 copies). Unlike miR-9-3p, data from the sTBI patients did not suggest any injury-dose effect. Two mTBI patients had both miR-9-3p and miR-136-3p levels higher than mean+1SD of that in controls, as well as plasma S100B levels higher than the reference of 0.1 µg/l (solid green diamonds). Statistical test: (Mann-Whitney's *U* test). Abbreviations: C, age-matched controls; mTBI, mild traumatic brain injury; PCR, polymerase chain reaction; sTBI, severe traumatic brain injury. Symbol key: Open pink circles indicate the controls age-matched with the 2 sTBI patients; black solid diamond indicates the mTBI patient with plasma S100B level <0.1 µg/l, but plasma miR-9-3p and miR-136-3p levels > mean+1SD of the controls; green solid diamonds indicate the mTBI patients with plasma S100B level >0.1 µg/l as well as miR-9-3p and miR-136-3p levels > mean+1SD of the controls; open green diamond indicates the mTBI patient with plasma S100B level >0.1 µg/l, but only miR-9-3p level > mean+1SD of the controls.

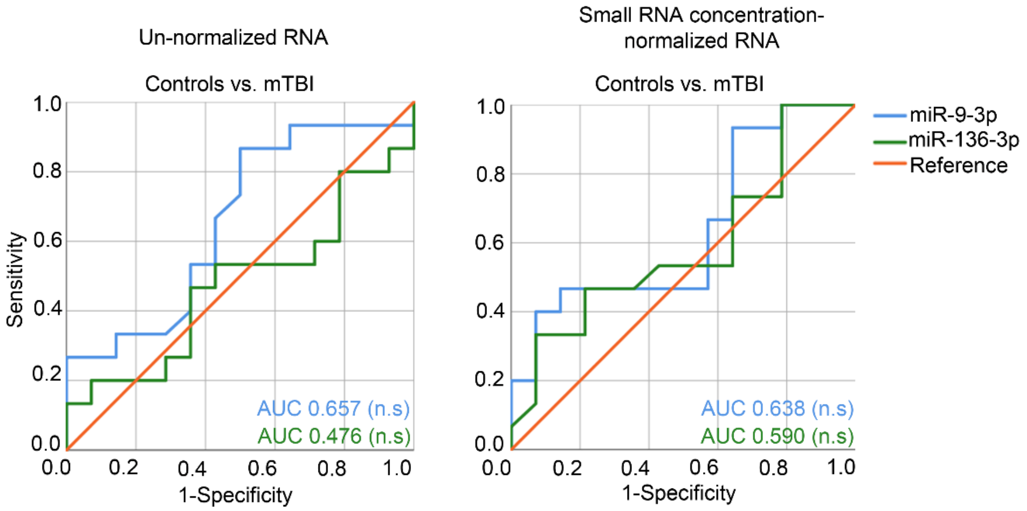

Supplementary Figure S6. Receiver operating characteristic (ROC) analysis indicated that plasma miR-9-3p and miR-136-3p levels did not distinguish patients with mTBI from controls. (A) Absolute copy numbers measured with ddPCR from un-normalized RNA indicated an area under the curve (AUC) of 0.657 (p>0.05) for miR-9-3p and 0.476 (p>0.05) for miR-136-3p. (B) When ddPCR was performed from the small RNA concentration-normalized RNA, miR-9-3p had an AUC of 0.638 (p>0.05) and miR-136 had an AUC of 0.590 (p>0.05). The combination of miR-9-3p and miR-136-3p slightly improved the AUC, but the increase was not significant. Abbreviations: AUC, area under the curve; mTBI, mild traumatic brain injury; n.s., not significant.

Supplementary Table 1. Receiver operator characteristic (ROC) curve and cut-point analysis for plasma rno-miR-9a-3p, rno-miR-136-3p and rno-miR-434-3p copy numbers in the whole animal cohort.

**rno-miR-9a-3p**				
**Comparisons**	**Area under the curve (AUC)**	**Cut-point (miRNA copy number)**	**Sensitivity (%)**	**Specificity (%)**
Naïve vs. Sham	0.525 (n.s)	-	-	-
Naïve vs. mTBI	1.000**	7	100	100
Naïve vs. sTBI	1.000**	64	100	100
Sham vs. mTBI	0.975**	12	90	100
Sham vs. sTBI	1.000**	64	100	100
mTBI vs. sTBI	1.000***	64	100	100
**rno-miR-136-3p**				
**Comparisons**	**Area under the curve (AUC)**	**Cut-point (miRNA copy number)**	**Sensitivity (%)**	**Specificity (%)**
Naïve vs. Sham	0.825 (n.s)	-	-	-
Naïve vs. mTBI	1.000**	10	100	100
Naïve vs. sTBI	1.000**	18	100	100
Sham vs. mTBI	0.712 (n.s)	-	-	-
Sham vs. sTBI	0.953**	18	100	88
mTBI vs. sTBI	0.913**	26	75	100
**rno-miR-434-3p**				
**Comparisons**	**Area under the curve (AUC)**	**Cut-point (miRNA copy number)**	**Sensitivity (%)**	**Specificity (%)**
Naïve vs. Sham	0.900*	13	75	100
Naïve vs. mTBI	1.000**	12	100	100
Naïve vs. sTBI	1.000**	53	100	100
Sham vs. mTBI	0.850*	20	90	75
Sham vs. sTBI	1.000**	53	100	100
mTBI vs. sTBI	1.000***	53	100	100

Abbreviations: mTBI, mild traumatic brain injury; Naïve, naïve rats; n.s, not significant; Sham, sham-operated experimental controls; sTBI, severe traumatic brain injury.

Statistical significances: *, $p<0.05$; **, $p<0.01$; ***, $p<0.001$.
